# Psychotropic drug-induced adverse drug reactions in 462,661 psychiatric inpatients in relation to age: results from a German drug surveillance program from 1993–2016

**DOI:** 10.1186/s12991-024-00530-0

**Published:** 2024-11-18

**Authors:** Johanna Seifert, Matthias A. Reinhard, Stefan Bleich, Andreas Erfurth, Waldemar Greil, Sermin Toto, Renate Grohmann, Catherine Glocker

**Affiliations:** 1 Department of Psychiatry, Social Psychiatry and Psychotherapy, Hannover Medical School, Carl-Neuberg-Straße 1, 30625 Hannover, Germany; 2 Department of Psychiatry and Psychotherapy, Ludwig Maximilian University, Munich, Germany; 3 1st Department of Psychiatry and Psychotherapeutic Medicine, Klinik Hietzing, Vienna, Austria; 4 Medical University of Vienna, Vienna, Austria; 5 Psychiatric Private Hospital, Sanatorium Kilchberg, Zurich, Kilchberg Switzerland

**Keywords:** Geriatric psychiatry, Aged, Drug-related side effects and adverse drug reactions, Drug safety, Pharmacovigilance, Polypharmacy

## Abstract

**Background:**

Clinical practice suggests that older adults (i.e., ≥ 65 years of age) experience adverse drug reactions (ADRs) more often than younger patients (i.e., < 65 years of age). ADRs such as falls, extrapyramidal symptoms (EPS), metabolic disorders, sedation, and delirium are particularly worrisome and often associated with psychotropic drugs.

**Methods:**

This observational study investigated the risk for psychotropic drug-related ADRs in older (n = 99,099) and younger adults (n = 363,562) in psychiatric inpatients using data from the German pharmacovigilance program “Arzneimittelsicherheit in der Psychiatrie” (AMSP) from 1993–2016. The aim was to assess whether age influenced the risk of specific ADR types and if certain psychotropic drugs posed particular concerns.

**Results:**

The risk for ADRs did not differ between older and younger patients (relative risk 0.98, 95% confidence interval 0.92–1.05). However, older patients had a higher risk for delirium (2.35, 1.85–2.99), hyponatremia (3.74, 2.85–4.90), and orthostatic syncope (2.37, 1.72–3.26), as well as certain types of EPS, e.g., parkinsonism (1.89, 1.45–2.48) and Pisa-/metronome syndrome (3.61, 2.51–5.18). The risk for other ADRs, such as acute dystonia (0.20, 0.10–0.37), akathisia (0.47, 0.29–0.76), liver dysfunction (0.63, 0.48–0.82), weight gain (0.07, 0.04–0.14), sexual dysfunction (0.03, CI 0.00–0.25), and hyperprolactinemia/galactorrhea (0.05, 0.02–0.17) was significantly lower for older patients. Older patients treated with any type of antidepressant drug (1.33, 1.26–1.40)—especially selective serotonin reuptake inhibitors (1.57, 1.26–1.40) and selective serotonin-norepinephrine reuptake inhibitors (2.03, 1.80–2.29)—and lithium (1.74, 1.52–2.00) had a higher ADR risk than younger patients. Second-generation antipsychotic drugs had a lower (0.74, 0.71–0.77) and low-potency first-generation antipsychotic drugs a higher (1.19, 1.07–1.33) ADR risk in older patients. The risk for ADRs involving multiple drugs was higher in older patients (1.28, 1.22–1.34). ADRs in older patients were 6.4 times more likely to result in death.

**Conclusions:**

Clinicians and pharmacists should be aware of the types of ADRs and high-risk drugs across age groups and provide appropriate monitoring. Pharmacovigilance is crucial in psychiatric patients of all ages and should not be neglected, even for drugs generally considered “safe”.

**Supplementary Information:**

The online version contains supplementary material available at 10.1186/s12991-024-00530-0.

## Introduction

The global population is aging rapidly, with projections from the World Health Organization indicating that by 2050, about 30% of the population will be comprised of adults aged ≥ 60 years. This demographic shift constitutes rising healthcare costs [1]. Contributing to these increased costs is the higher vulnerability of older adults to drug-related morbidity and mortality due to a higher burden of chronic disease, side effects of polypharmacy (defined as the use of 5 or more drugs [2]), and age-related physiological changes in drug metabolism [3]. Psychotropic drugs warrant particular attention in this context. This concern aligns with both the recently revised German PRISCUS List, which classifies nearly all psychotropic drugs as “potentially inappropriate medication” when used in patients aged ≥ 65 years of age [4] and the American Beer’s Criteria that also recommend a prudent use of psychotropic drugs in older patients [5]. Indeed, psychotropic drug use in older adults is significantly associated with adverse health outcomes, such as hospitalization [6] and falls [7]. Another important aspect is the occurrence of adverse drug reactions (ADRs), defined as unpleasant or potentially harmful reactions to a drug necessitating specific treatment, dose reduction, or drug withdrawal [8]. Not only are psychotropic drugs frequently associated with the occurrence of ADRs [9], psychotropic drug-related ADRs are often considered preventable [10].

Pharmacovigilance plays an important role in monitoring the safety of drugs. In fact, in the European Union, physicians and pharmacists are required to report ADRs to the respective national institutions, such as the German Federal Institute for Drugs and Medical Devices (“Bundesinstitut für Arzneimittel und Medizinprodukte”, BfArM). The advantage of sponateous reporting systems is that they collect data from a wide range of healthcare providers, in some cases even directly from patients. They are essential for detecting rare and unusual ADRs and, in contrast to randomized controlled trials (RCTs), they do not have any exclusion criteria, therefore offering an assessment of ADRs in an uncontrolled, “real-life” setting [11].

ADRs are a major health concern that affect 5–60% of older hospitalized inpatients, [12]. Heck et al. examined the prevalence of ADRs in geriatric psychiatric inpatients over a 6-year period, determining an overall ADR prevalence of 8.8%. The most common ADRs were extrapyramidal symptoms (EPS), cardiovascular symptoms, and electrolyte disturbances, however, the authors included ADRs associated with any type of drug [13]. A previous study using data from the project “Drug Safety in Psychiatry” (German: “Arzneimittelsicherheit in der Psychiatrie e.V.”; AMSP), which included 39,728 inpatients and 699 severe ADRs from 2001–2010 in Switzerland, found an inverse correlation between patient age and the occurrence of psychotropic drug-induced ADRs, particularly weight gain, EPS, galactorrhea, and elevated transaminases [14]. Other studies examining the incidence of psychotropic drug-induced ADRs using AMSP data support these findings [15–17]. Thus, it appears that occurrence of several common psychotropic drug-associated ADRs show age-dependent effects. Some types of ADRs are more likely to affect younger patients (e.g., weight gain [15], galactorrhea [17], drug-induced liver injury [16]), while older patients are at higher risk for others (e.g., delirium [18], hyponatremia [19]).

The objective of the present study is to comprehensively analyze the risk of psychotropic drug-induced ADRs in patients ≥ 65 years of age compared to younger patients (i.e., < 65 years of age). We sought to determine (a) which types of ADRs had a higher risk in older versus younger adults and (b) which psychotropic drugs and drug groups are of particular concern. We hypothesized that the risk for several ADRs, such as weight gain and galactorrhea, are more common in younger patients, while the risk for EPS, hyponatremia, and delirium is higher in older patients. Further, we suspected that drugs with strong anticholinergic properties are associated with a higher risk of ADRs in older patients. This data holds considerable value in clinical practice as it aids in evaluating the association between particular drugs and their long-term risks, especially in the extended treatment of both younger and elderly patients.

## Methods

### The AMSP program and data collection

Aiming to improve pharmacovigilance in the field of psychiatry, the AMSP program was established in 1993 in German-speaking countries (i.e., Germany, Austria, Switzerland). AMSP is an ongoing project that monitors drug safety in a “real life” psychiatric inpatient setting. The AMSP database consists of two distinct sets of data. The first dataset comprises pharmacoepidemiologic data, i.e., drug use data, age, and sex of all patients treated in the hospitals participating in the AMSP project at the time of data collection. This data is collected on two index days per year. In addition, information on the number of patients monitored each year and the average duration of inpatient stay allows an estimation of the number of patients exposed to a particular drug/combination of drugs. The second dataset contains events of severe, unusual, and/or rare ADRs (see below) occurring in psychiatric inpatients during treatment with psychotropic drugs.

An ADR is defined as any adverse event occurring at doses adequate for therapeutic or prophylactic treatment. This does not include adverse events due to intoxication or inefficiency. ADRs are classified according to affected organ systems (e.g., psychiatric, neurological, cardiovascular). The AMSP manual includes guidelines for determining the severity of the ADR, allowing a standardized assessment of ADRs [20]. This study includes only events of ADRs classified as “severe”. All data collected is anonymized.

### Assessment and collection of ADRs

Data on ADRs is collected by psychiatrists designated as drug monitors. Drug monitors regularly (i.e., at least bi-weekly) consult with treating physicians on psychiatric wards about the occurrence of ADRs in patients. ADRs are documented using a standardized questionnaire and carefully reviewed for plausibility by a senior physician. The causal relationship between an ADR and the implicated drug(s) is classified as ‘possible’, ‘probable’, ‘definite’, or ‘questionable’ according to AMSP standards [20]:

- Grade 1: possible (ADR unknown, alternative explanation more likely)
- Grade 2: probable (ADR known for drug imputed, time of onset and dose are plausible; alternative explanation less likely)
- Grade 3: definite (same as 2 with reoccurrence of the ADR after re-exposure with the drug imputed)
- Grade 4: questionable or insufficient documentation

Only ADRs with a probability rating of ‘probable’ or ‘definite’ were considered in this study. An ADR is viable for inclusion in the AMSP database, (1) if it is either considered “severe” (i.e., it is [potentially] life-threatening or seriously endangers a patient’s health, (2) if it causes considerable impairment of everyday functioning, or (3) if it necessitates a transfer to another ward or department for more specialized care). The AMSP manual includes detailed guidelines for determining the severity of ADRs, allowing a standardized assessment of ADRs [20, 21].

The present study includes ADR reports from 107 hospitals that participated in the AMSP program during the study period. Most ADRs (72.4%) stem from a total of 71 German hospitals, 17.2% from Switzerland (22 hospitals), 9.9% from Austria (12 hospitals), and 0.5% from Hungary and Belgium (1 hospital each). Of note, the present study also includes the ADRs using only Swiss AMSP data analyzed in the study by Greil et al. [14].

Because many patients are treated with multiple drugs, more than one drug may be implicated in the ADR in question. This can be due either to direct effects of the other drug(s) causing the same ADR or through pharmacokinetic interactions. When multiple drugs are implicated in an ADR, the causal relationship of each drug is evaluated individually. Therefore, AMSP distinguishes between three subgroups of ADR cases: cases in which only one drug was implicated (i.e., “single imputation”), cases in which a combination of drugs was imputed (i.e., “multiple imputation”), and “all cases”, which includes both of the above mentioned [20].

### Classification of psychotropic drugs relevant to the present study

A classification of psychotropic drugs most commonly used in this study’s patient collective (i.e., drugs used in ≥ 1.0% of patients) can be found in the supplementary material (suppl. Table 1).

### Inclusion criteria of the present study and definition of “older” and “younger” patients

The present study includes patients monitored by the AMSP Program from 1993 to 2016. Primary psychiatric diagnosis is presented according to the International Classification of Disease, 10th Version (ICD-10). We only included patients who were treated with psychotropic drugs (N = 462,661), as those without drug use aren’t at risk for ADRs. We defined “older patients” as those aged ≥ 65 years because this is the most commonly used age limit in scientific research and guidelines statements [22]. Accordingly, the term “younger patients” refers to those aged 18–65 years.

### Statistical methods

The main objective of the present study was to determine (a) the risk of different types of ADRs and (b) the risk for ADR of different types of psychotropic in older vs. younger patients. The incidence of ADRs was calucluated in percent of patients exposed to psychotropic drugs or a specific psychotropic drug/drug class. The risk of different types of ADRs, as well as the risk for ADRs associated with different psychotropic drugs for older vs. younger patients, was calculated as relative risks (RRs) including their respective 95% confidence intervals (CIs). RRs were also used to determine the general risk of ADRs according to age and diagnostic group in the two age groups. A RR > 1 implies a higher ADR-risk for older vs. younger patients, while a RR < 1 implies a lower ADR-risk for older patients.

Chi-squared tests were used to compare categorical characteristics (i.e., sex, diagnosis) of the collective in older and younger patients (N = 462,661), as well as countermeasures taken in ADRs (N = 5729). The mean number (± standard deviation [SD]) of different types of (psychotropic) drugs in different patient groups were calculated. The Shapiro–Wilk test was used to assess normality. As data were not consistently normally distributed, unpaired t-tests were used to determine statistical significances. Cohen’s *d* was calculated as measure of effect size (*d* = 0.2, small; *d* = 0.5, medium; *d* = 0.8, large). All statistics were performed using Excel^©^ and SPSS^©^ version 26 by IBM. The significance level was set at *p* < 0.05.

## Results

### Characteristics of the study population

#### Characteristics according to age group

Between 1993 and 2016, the AMSP program monitored a total of 462,661 psychiatric inpatients who were treated with at least one psychotropic drug. 99,099 patients were aged ≥ 65 years (21.4% of all patients). The median age in the group of older patients was 75.0 years and 40.7 years in the younger group of patients. The proportion of females was significantly higher among older patients than among those aged < 65 years (68.3% vs. 52.5%;). Older patients suffered from organic (33.0% vs. 6.5%) and depressive disorders (41.1% vs. 32.3%) significantly more often and were significantly less likely to suffer from substance-related disorders (2.2% vs. 5.1%), schizophrenia (16.1% vs. 39.1%), and acute mania (2.4% vs. 3.0%; Table 1). Older patients were treated with an average of 5.37 ± 2.58 drugs compared to 3.50 ± 2.10 drugs in younger patients (*p* < 0.001, *d* = 0.845). The difference in the mean number of psychotropic drugs, antidepressant drugs (ADDs), and antipsychotic drugs (APDs) between age groups were either statistically insignificant or of small effect size (suppl. Table 2A).

**Table 1:** Characteristics (i.e., sex and diagnosis) of the study population according to age group (≥ 65 vs. < 65 years)

	Patients ≥ 65 years (% of patients ≥ 65 years)	Patients < 65 years (% of patients < 65 years)	Chi^2^-Test (χ^2^, df, *p*)	Post-hoc Chi^2^ (χ^2^, df, *p*)
Total	99,099 (100%)	363,562 (100%)		
Sex				
Females	67,655 (68.3%)	190,935 (52.5%)	χ^2^ = 7837.955; df = 1; *p* < 0.001	
Males	31,444 (31.7%)	172,627 (47.5%)		
Diagnosis (ICD-10)				
Organic disorders (F0)^a^	32,677 (33.0%)	23,742 (6.5%)	χ^2^ = 64,910.712; df = 5; *p* < 0.001	χ^2^ = 50,856.358; df = 1; *p* < 0.001
Substance-related disorders (F1)	2217 (2.2%)	18,420 (5.1%)		χ^2^ = 1462.853; df = 1; *p* < 0.001
Schizophrenia (F2)	15,940 (16.1%)	142,097 (39.1%)		χ^2^ = 18,316.130; df = 1; *p* < 0.001
Depressive disorders (F3 without F30, F31.0–F31.2)	40,685 (41.1%)	117,313 (32.3%)		χ^2^ = 2673.889; df = 1; *p* < 0.001
Acute mania (F30, F31.0–F31.2)	2344 (2.4%)	10,823 (3.0%)		χ^2^ = 105.358; df = 1; *p* < 0.001
Others (F4–F9)	5236 (5.3%)	51,167 (14.1%)		χ^2^ = 5620.874; df = 1; *p* < 0.001

*df* degrees of freedom; *ICD-10* International Classification of Disease, 10th Version

^a^Including organic disorders from F1 and F7

#### Relative risk for adverse drug reactions according to sex and diagnosis

A total of 5729 patients experienced severe ADRs (1.24% of 462,661). The overall risk for ADRs did not differ between older and younger patients (RR 0.98, CI 0.95–1.02). However, older women had a significantly higher risk for ADRs than younger females (RR 1.84, CI 1.76–1.92), while older men had a significantly lower risk for ADRs than younger men (RR 0.65, CI 0.62–0.68). Older patients with depressive disorders had a significantly higher risk of ADRs than younger patients with this diagnosis (RR 1.29, CI 1.22–1.36), whereas older patients with schizophrenia (RR 0.73, CI 0.69–0.77) and acute mania (RR 0.69, CI 0.60–0.79) had a lower risk of ADRs than younger patients (Table 2). Older patients with ADRs were treated with an average of 5.45 ± 2.59 drugs compared to 3.49 ± 2.00 drugs in younger patients (*p* < 0.001, *d* = 0.907). The difference in the mean number of psychotropic drugs, ADDs, and APDs between age groups were either statistically insignificant or of small effect size (suppl. Table 2B).

**Table 2:** Relative risk for adverse drug reactions of patients according to sex and diagnosis in patients ≥ 65 vs. < 65 years

	Patients ≥ 65 years of age			Patients < 65 years of age			≥ 65 vs. < 65
	N patients with ADR	N patients (all)	% of patients with ADR	N patients with ADR	N patients (all)	% of patients with ADR	RR (95% CI)
Total	1212	99,099	1.22%	4517	363,562	1.24%	0.98 (0.95–1.02)
Sex							
Females	863	67,655	1.28%	2020	290,935	0.69%	1.84 (1.76–1.92)
Males	349	31,444	1.11%	2949	172,627	1.71%	0.65 (0.62–0.68)
Diagnosis (ICD-10)							
Organic disorders (F0)	337	32,677	1.03%	242	23,732	1.02%	1.01 (0.89–1.15)
Substance-related disorders (F1)	14	2217	0.63%	136	18,420	0.74%	0.86 (0.72–1.02)
Schizophrenia (F2)*	170	15,940	1.07%	2078	142,097	1.46%	0.73 (0.69–0.77)
Depressive disorders (F3 without F30, F31.0–F31.2)*	626	40,685	1.54%	1402	117,313	1.20%	1.29 (1.22–1.36)
Acute mania (F30, F31.0–F31.2)*	36	2344	1.54%	241	10,823	2.23%	0.69 (0.60–0.79)
Others (F4–F9)*	29	5236	0.55%	385	51,167	0.75%	0.74 (0.66–0.82)

*ADR* adverse drug reaction; *N* number (of); *RR* relative risk; *CI* confidence interval; *ICD-10* International Classification of Disease, 10th Version

^*^Indicates a significant finding

### Type of psychotropic drug-induced adverse drug reactions according to age group

#### All imputations (i.e., single and multiple imputation)

**Fig. 1:**
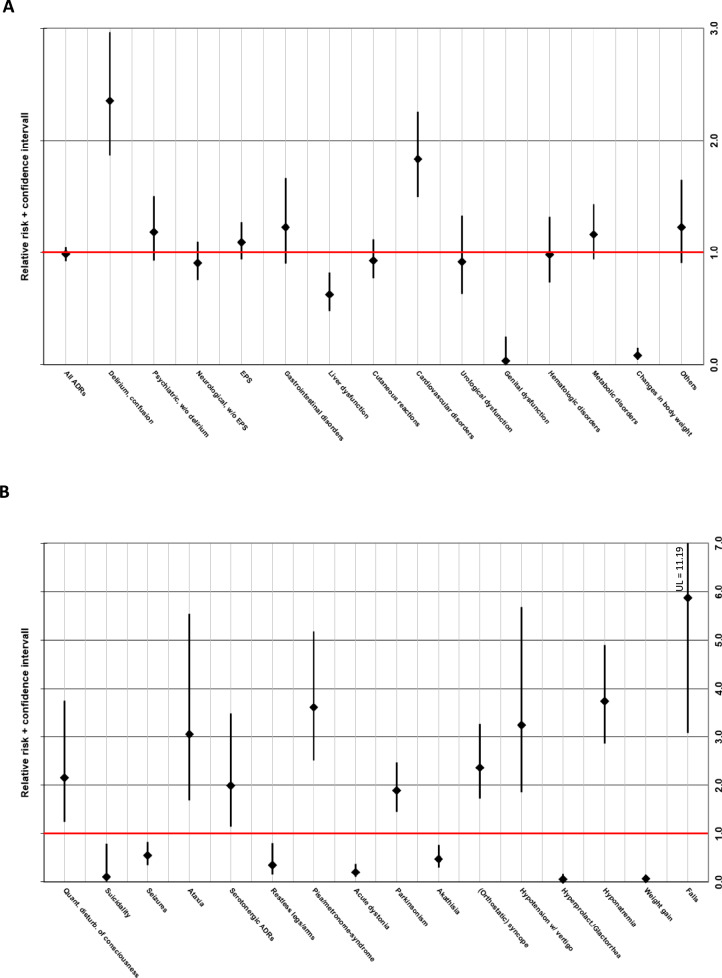
Relative risk (RR) incl. 95% confidence interval of (**A**) adverse drug reactions (ADRs) affecting different organ systems and (**B**) a selection* of individual ADRs (all imputations) in older vs. younger patients. RR > 1 implies a higher ADR-risk for older patients compared to younger patients; RR < 1 implies a lower ADR-risk for older patients compared to younger patients. *selection is based on ADRs for which we detected significant differences between the two age groups. *w/* with; *w/o* without; *EPS* extrapyramidal symptoms

Figure 1A shows the RR of ADRs according to the affected organ system and age group, while Fig. 1B depicts the RR of a selection of individual ADRs that showed significant differences between the two age groups. Table 3 shows the RR (including 95% CI) of the affected organ systems, as well as of the frequent individual ADRs (cut-off ≥ 35 cases among all patients) for all cases (i.e., single and multiple imputations).

**Table 3:** Incidence and relative risk of different types of adverse drug reactions (all imputations) in older vs. younger patients

Adverse drug reaction	Patients ≥ 65 years of age (N = 99,099)		Patients < 65 years of age (N = 363,562)		≥ 65 vs. < 65
	N cases	% of patients	N cases	% of patients	RR (95% CI)
**All ADRs**	1212	1.223	4517	1.242	0.98 (0.92–1.05)
Delirium, confusion*	118	0.119	184	0.051	2.35 (1.87–2.96)
Delirium*	109	0.110	170	0.047	2.35 (1.85–2.99)
**Psychiatric symptoms, excl. delirium**	87	0.088	270	0.074	1.18 (0.93–1.51)
Disturbance of consciousness*	20	0.020	34	0.009	2.16 (1.24–3.75)
Psychosis/(pseudo-) hallucinations	9	0.009	39	0.011	0.85 (0.41–1.75)
Restlessness/agitation	24	0.024	66	0.018	1.33 (0.84–2.13)
Sedation	15	0.015	37	0.010	1.49 (0.82–2.71)
Suicidality*	1	0.001	34	0.009	0.11 (0.01–0.79)
**Neurological symptoms, excl. EPS**	132	0.133	533	0.147	0.91 (0.75–1.10)
Seizures*	24	0.024	162	0.045	0.54 (0.35–0.83)
Myoclonus	6	0.006	35	0.010	0.63 (0.26–1.50)
Ataxia*	20	0.020	24	0.007	3.06 (1.69–5.53)
Tremor	20	0.020	62	0.017	1.18 (0.71–1.96)
Vision disorders, glaucoma	5	0.005	30	0.008	0.61 (0.24–1.58)
Serotonin-syndrome, serotonergic ADRs*	19	0.019	35	0.010	1.99 (1.14–3.48)
Restless legs/arms*	6	0.006	63	0.017	0.35 (0.15–0.81)
**EPS**	212	0.214	712	0.196	1.09 (0.94–1.27)
Neuroleptic malignant syndrome	13	0.011	42	0.012	1.14 (0.61–2.12)
Tardive dyskinesia	8	0.008	45	0.012	0.65 (0.31–1.38)
Pisa/metronome-syndrome*	58	0.059	59	0.016	3.61 (2.51–5.18)
Atypical dyskinesia	15	0.015	55	0.015	1.00 (0.57–1.77)
Acute dystonia*	10	0.010	185	0.051	0.20 (0.10–0.37)
Parkinsonism*	80	0.081	155	0.043	1.89 (1.45–2.48)
Akathisia*	19	0.019	147	0.040	0.47 (0.29–0.76)
**Gastrointestinal disorders**	54	0.054	162	0.045	1.22 (0.90–1.66)
(Sub)ileus/severe constipation	11	0.011	38	0.010	1.06 (0.54–2.08)
Nausea/vomiting	13	0.013	36	0.010	1.32 (0.70–2.50)
Liver dysfunction*	61	0.062	358	0.098	0.63 (0.48–0.82)
Elevated transaminases*	61	0.062	356	0.098	0.63 (0.48–0.82)
**Cutaneous reactions**	137	0.138	541	0.149	0.93 (0.77–1.12)
Edema	49	0.049	182	0.050	0.99 (0.72–1.35)
Allergic cutaneous reactions	80	0.081	313	0.086	0.94 (0.73–1.20)
Cardiovascular disorders*	135	0.136	270	0.074	1.83 (1.49–2.26)
(Orthostatic) syncope*	62	0.063	96	0.026	2.37 (1.72–3.26)
Symptomatic hypotension with vertigo*	23	0.023	26	0.007	3.25 (1.85–5.69)
Arrhythmia	24	0.024	77	0.021	1.14 (0.72–1.81)
Prolonged QT-interval	10	0.010	32	0.009	1.15 (0.56–2.33)
**Urological dysfunction**	35	0.035	140	0.039	0.92 (0.63–1.33)
Urinary retention	27	0.027	97	0.027	1.02 (0.67–1.56)
Genital dysfunction*	1	0.001	106	0.029	0.03 (0.00–0.25)
Erectile dysfunction	0	0.000	65	0.018	–
**Hematologic disorders**	56	0.057	209	0.057	0.98 (0.73–1.32)
Agranulocytosis	12	0.012	43	0.012	1.02 (0.54–1.94)
Neutropenia	18	0.018	89	0.024	0.74 (0.45–1.23)
Thrombocytopenia	9	0.009	30	0.008	1.10 (0.52–2.32)
**Metabolic disorders, electrolyte imbalances**	115	0.116	364	0.100	1.16 (0.94–1.43)
Hyponatremia*	106	0.107	104	0.029	3.74 (2.85–4.90)
Increased prolactin/galactorrhea*	3	0.003	206	0.057	0.05 (0.02–0.17)
Changes in body weight*	11	0.011	494	0.136	0.08 (0.04–0.15)
Weight gain*	10	0.010	493	0.136	0.07 (0.04–0.14)
**Others**	58	0.059	174	0.048	1.22 (0.91–1.65)
Falls*	24	0.024	15	0.004	5.87 (3.08–11.19)

*N* number (of); *RR* relative risk; *CI* confidence interval; *ADR* adverse drug reaction; *EPS* extrapyramidal symptoms

^*^Indicates a significant result

Compared to younger patients, older patients had a 2.35-fold (CI 1.87–2.96) higher risk of experiencing “delirium and confusion”. However, older patients had a significantly lower risk for drug-induced suicidality than younger patients (RR 0.11, CI 0.01–0.79; Fig. 1A; Table 3).

In general, the risk of neurological symptoms (Fig. 1A; Table 3) did not differ between age groups. But while older patients were less likely to experience seizures (RR 0.54, CI 0.35–0.83) and restless legs/arms (RR 0.35, CI 0.15–0.81), their risk for ataxia (RR 3.06, CI 1.69–5.53) and serotonergic ARDs (RR 1.99, CI 1.14–3.48) was significantly higher compared to younger patients. Similarly, while the risk of EPS in general did not show age-dependent effects, the risk for several types of EPS, such as parkinsonism (RR 1.89, CI 2.45–2.48) and Pisa/metronome-syndrome (RR 3.61, CI 2.51–5.18), was significantly higher in older patients. On the other hand, older patients had a significantly lower risk for acute dystonia (RR 0.20, CI 0.10–0.37) and akathisia (RR 0.47, CI 0.29–0.76; Fig. 1B; Table 3).

ADRs affecting the cardiovascular system were 1.83 times (CI 1.49–2.26) more likely in older patients (Fig. 1A; Table 3). In particular, older patients had a significantly higher risk of (orthostatic) syncope (RR 2.37, CI 1.72–3.26) and hypotension with vertigo (RR 3.25, CI 1.85–5.69; Fig. 1B; Table 3).

The risk of liver dysfunction (mainly elevated transaminases; RR 0.63, CI 0.48–0.82), changes in body weight (almost exclusively weight gain; RR 0.08, CI 0.04–0.15), and genital disorders (mainly including different types of sexual dysfunction; RR 0.03, CI 0.00–0.25; Fig. 1A; Table 3) was significantly lower among older patients. Older patients had a 3.74fold higher risk of psychotropic drug-induced hyponatremia (CI 2.85–4.90), while the risk for symptomatic hyperprolactinemia and galactorrhea (RR 0.05, CI 0.02–0.17) was significantly lower in older patients (Fig. 1B; Table 3). Lastly, older patients had a 5.87fold higher risk of experiencing psychotropic drug-related falls (CI 3.08–11.19; Fig. 1B; Table 3).

#### Single and multiple imputations

**Fig. 2:**
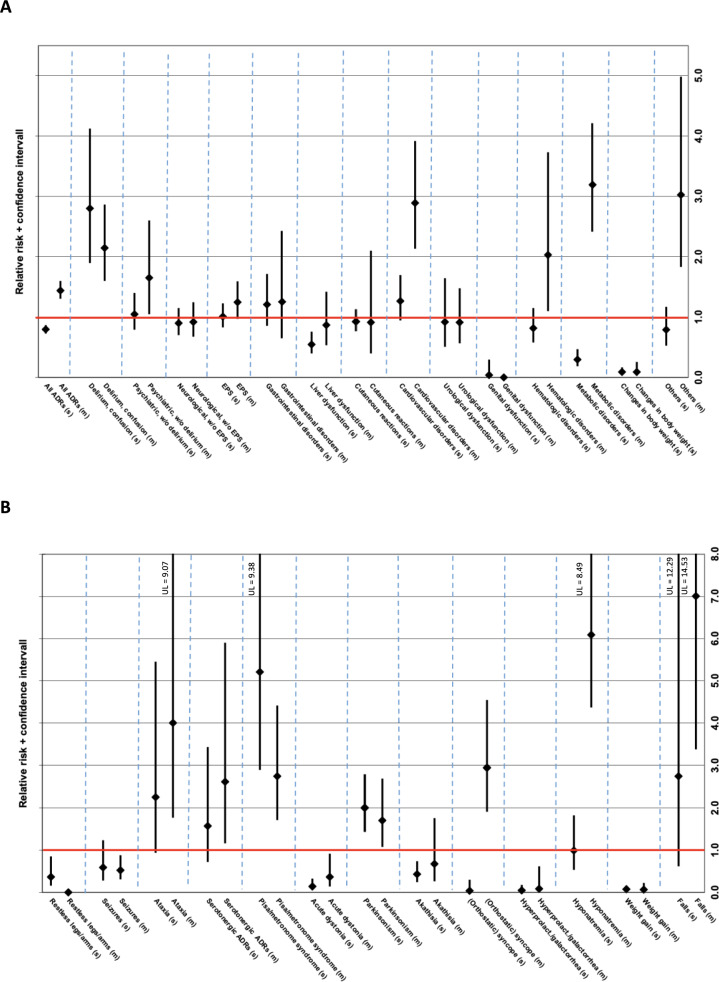
Relative risk (RR) incl. 95% confidence interval of single vs. multiple imputation adverse drug reactions (ADRs) (**A**) according to the affected organ system and (**B**) in a selection* of individual ADRs in older vs. younger patients. RR > 1 implies a higher ADR-risk for older patients compared to younger patients; RR < 1 implies a lower ADR-risk for older patients compared to younger patients. *selection is based on ADRs for which we detected significant differences between the two age groups. *(s)* single imputation ADR; *(m)* multiple imputation ADR; *w/* with; *w/o* without; *EPS* extrapyramidal symptoms; *UL* upper limit

Figure 2A shows the RR of single vs. multiple imputation ADRs according to the affected organ system and age group, while Figs. 2B depicts the RR of single vs. multiple imputation in a selection of individual ADRs that showed significant differences between the two age groups. Tables with the RR for all single (Suppl. Table 3) and multiple imputation ADRs (Suppl. Table 4) can be found in the supplementary material.

3212 of 4517 ADRs (71.1% of all ADRs in younger patients) affecting younger patients implicated a single drug, while in older patients, 698 of 1212 ADRs (57.6% of all ADRs in older patients) implicated a single drug. Overall, the risk for multiple imputation ADRs was significantly higher in older than in younger patients (RR 1.44, CI 1.30–1.60; Fig. 2A; suppl. Table 4). Among organ systems, the risk for multiple imputation ADRs in older patients was significantly higher for psychiatric symptoms (excluding delirium; RR 1.65, CI 1.05–2.60), cardiovascular disorders (RR 2.89, CI 2.13–3.92), hematologic disorders (RR 2.02, CI 1.10–3.73), and metabolic disorders (RR 3.19, CI 2.41–4.21 Fig. 2A; suppl. Table 4). The risk for “delirium and confusion” was higher in older than in younger patients as a single imputation (RR 2.80, CI 1.90–4.12; Fig. 2A; suppl. Table 3), as well as a multiple imputation ADR (RR 2.14, CI 1.61–2.86; Fig. 2A; suppl. Table 4).

Among individual ADRs, older patients had a particularly high risk for (orthostatic) syncope (RR 2.95, CI 1.91–4.55) and hyponatremia (RR 6.09, CI 4.37–8.49) imputing multiple drugs compared to younger patients (Fig. 2B; suppl. Table 4). On the other hand, restless legs/arms, acute dystonia, hyperprolactinemia/glactorrhea, and weight gain had a significantly higher risk of affecting younger patients, both as single and as multiple imputation ADRs (Fig. 2B; suppl. Tables 3 and 4).

### Adverse drug reactions by psychotropic drug class and specific psychotropic drugs

#### Rate of adverse drug reactions under treatment with psychotropic drugs (subgroups) and individual psychotropic drugs according to age group

**Fig. 3:**
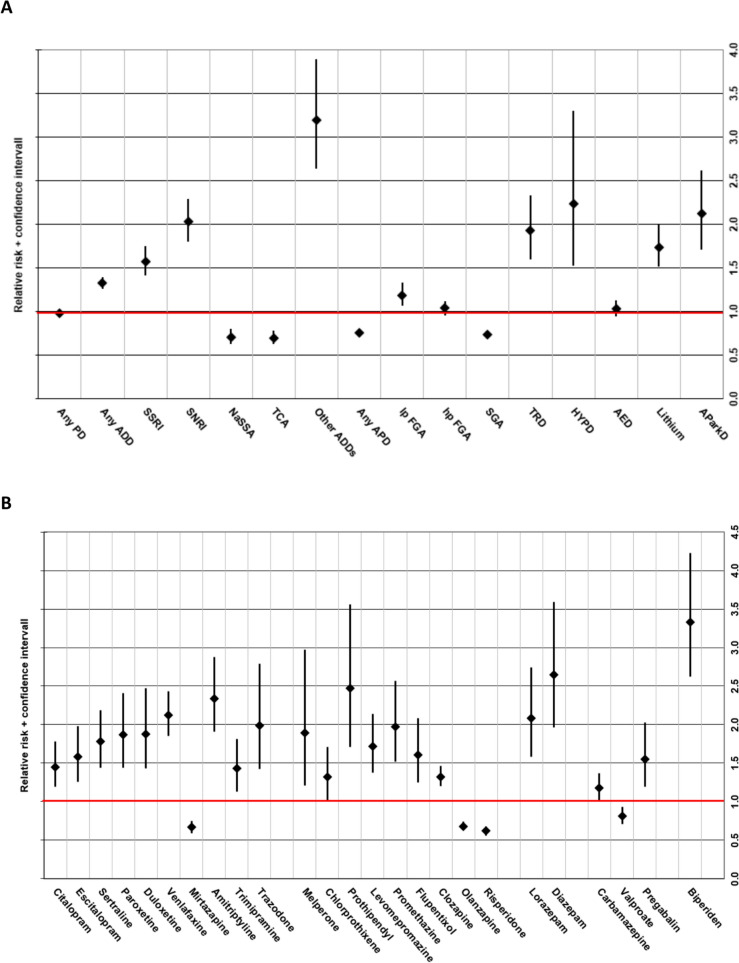
Relative risk (RR) incl. 95% confidence interval for ADRs (all imputations) of (**A**) different psychotropic drug classes and (**B**) in a selection* of different individual psychotropic drugs in older vs. younger patients. RR > 1 implies a higher ADR-risk for older patients compared to younger patients; RR < 1 implies a lower ADR-risk for older patients compared to younger patients. *selection is of psychotropic drugs for which we detected a significant relative risk. *PD* psychotropic drug; *ADD* antidepressant drug; *SSRI* selective serotonin reuptake inhibitor; *SNRI* selective serotonin-norepinephrine reuptake inhibitor; *TCA* tricyclic antidepressant; *NaSSA* noradrenergic and specific serotonergic antidepressant; *APD* antipsychotic drug; *FGA* “first-generation” antipsychotic drug; *lp* low potency; *hp* high potency; *SGA* “second-generation” antipsychotic drug; *HYPD* hypnotic drug; *TRD* tranquilizing drug; *AED* antiepileptic drug; *AParkD* antiparkinson drug

Figure 3 provides an overview of the RRs for ADRs of different types of psychotropic drugs groups and subgroups (Fig. 3A) and individual psychotropic drugs for which we detected a significant RR between the two age groups (Fig. 3B). The RRs shown include all events of ADRs (i.e., imputation of a single drug and multiple drugs). A table with all psychotropic drugs and their respective RR can be found in the supplementary material (suppl. Table 5).

##### Antidepressant drugs and lithium

Overall, older patients treated with ADDs had a significantly higher risk of experiencing ADRs than younger patients (RR 1.33, CI 1.26–1.40). This was observed for the two subgroups of selective serotonin reuptake inhibitors (SSRIs; RR 1.57, CI 1.42–1.75) and selective serotonin-norepinephrine reuptake inhibitors (SNRIs; RR 2.03, CI 1.80–2.29; Fig. 3A, suppl. Table 5). We found a higher RR for ADRs in older vs. younger patients for all individual SSRIs and SNRIs examined (Fig. 3B, suppl. Table 5). In general, the RR for ADRs in older patients treated with tricyclic antidepressants (TCAs) was significantly lower than in younger patients (RR 0.70, CI 0.63–0.80; Fig. 3A, suppl. Table 5). However, when considering individuals TCAs, older users of amitriptyline and trimipramine had a significantly higher risk for ADRs than younger patients (Fig. 3B, suppl. Table 5). The risk of ADRs among older patients treated with noradrenergic and specific serotonergic antidepressants (NaSSAs) was significantly lower than among younger patients (RR 0.71, CI 0.63–0.80). Older patients treated with lithium had a 1.74fold (CI 1.52–2.00) risk of ADRs compared to younger patients (Fig. 3A, suppl. Table 5).

##### Antipsychotic drugs

In general, older APD-users had a significantly lower risk of ADRs than younger APD-users (RR 0.76, CI 0.73–0.79; Fig. 3A, suppl. Table 5), however, the risk of ADRs of different subgroups of ADRs varied between the age groups. Low potency first-generation antipsychotic drugs (FGAs) had a higher risk for ADRs in older patients (RR 1.19, CI 1.07–1.33; Fig. 3A, suppl. Table 5), as was the case for melperone, chlorprothixene, prothipendyl, levomepromazine, and promethazine (Fig. 3B, suppl. Table 5). The ADR risk among hp FGA-users did not significantly differ between age groups (Fig. 3A, suppl. Table 5), which the exception of flupentixol (Fig. 3B, suppl. Table 5). While the overall ADR risk in older SGA users was lower than in younger SGA users (RR 0.74, CI 0.71–0.77; Fig. 3A, suppl. Table 5), older patients treated with clozapine had a significantly higher risk for ADRs and those treated with risperidone or olanzapine had a significantly lower risk for ADRs than younger patients (Fig. 3B, suppl. Table 5).

##### Tranquilizing and hypnotic drugs

Older patients treated with tranquilizing and hypnotic drugs had a significantly higher risk for ADRs than younger patients (RR 1.93, CI 1.60–2.33 resp. RR 2.24, CI 1.53–3-30; Fig. 3A, suppl. Table 5).

##### Antiparkinson drugs

The RR of ADRs under treatment with antiparkinson drugs among older patients was significantly higher than in younger patients (RR 2.12, CI 1.71–2.62; Fig. 3A, suppl. Table 5), especially for biperiden (RR 3.33, CI 2.62–4.23; Fig. 3B, suppl. Table 5).

#### Adverse drug reactions with imputation of a single vs. multiple drugs according to age group

**Fig. 4:**
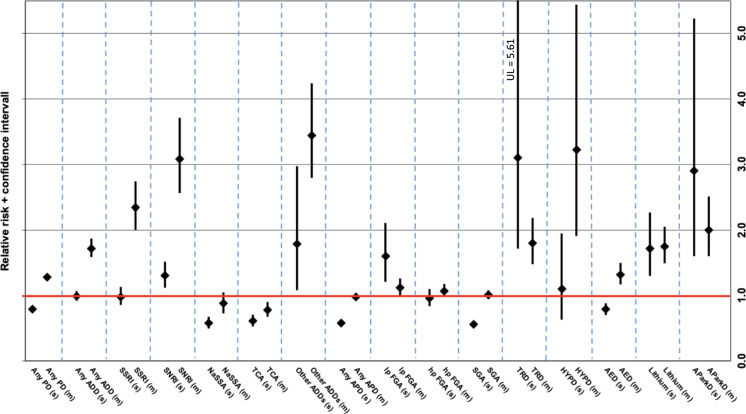
Relative risk (RR) incl. 95% confidence interval of single vs. multiple imputation adverse drug reactions (ADRs) for psychotropic drug groups/subgroups in older vs. younger patients. RR > 1 implies a higher ADR-risk for older patients compared to younger patients; RR < 1 implies a lower ADR-risk for older patients compared to younger patients. *(s)* single imputation ADR; *(m)* multiple imputation ADR; *PD* psychotropic drug; *ADD* antidepressant drug; *SSRI* selective serotonin reuptake inhibitor; *SSNRI* selective serotonin-norepinephrine reuptake inhibitor; *TCA* tricyclic antidepressant; *NaSSA* noradrenergic and specific serotonergic antidepressant; *APD* antipsychotic drug; *FGA* “first-generation” antipsychotic drug; *lp* low potency; *hp* high potency; *SGA* “second-generation” antipsychotic drug; *HYPD* hypnotic drug; *TRD* tranquilizing drug; *AED* antiepileptic drug; *AParkD* antiparkinson drug; *UL* upper limit

Figure 4 shows the RR for single and multiple imputation ADRs of different psychotropic drug groups. The exact RR and CIs can be found in the supplementary material (suppl. Table 6 for single imputation ADRs and suppl. Table 7 for multiple imputation ADRs).

The risk of multiple imputation ADRs in older adults was significantly higher than in younger patients (RR 1.28, CI 1.22–1.34, suppl. Table 7), as was the case for most drug groups. While the risk for SSRI-associated single imputation ADRs did not significantly differ between age groups (Fig. 4, suppl. Table 6), the risk for multiple imputation ADRs under SSRI was significantly increased for older patients compared to younger patients (Fig. 4, suppl. Table 7). For SNRI, on the other hand, risk of both single and multiple imputation ADRs was signifcantly higher for older patients. This was also the case for lithium, tranquilizing drugs, and antiparkinson drugs (Fig. 4, suppl. Tables 6 and 7). Among APDs, older patients had a significantly lower risk of single imputation ADRs (Fig. 4, suppl. Table 6), whereas the risk for multiple imputation ADRs did not differ from younger patients, as was the case for SGAs (Fig. 4, suppl. Table 7). However, low potency FGAs were the only drug class for which the risk of single imputation ADRs tended to be higher than for multiple imputation ADRs was low-potency FGAs. However, the confidence intervals overlap, indicating that this difference (i.e., that the risk of older patients for single imputation ADRs is higher than for multiple imputation ADRs) is not statistically significant (Fig. 4, suppl. Tables 6 and 7).

### Drug dosages in patients with and without ADRs according to age group

Table 4 provides information on median daily dosages of patients who suffered from an ADRs compared to all exposed patients according to age group. Younger patients were generally treated with higher doses than older patients. Exceptions were venlafaxine and mirtazapine, for which the daily doses did not differ between the two age groups. Pipamperone was the only drug that had the highest dosage in older patients suffering from an ADR. Compared within the respective age group, dosages were higher for ADR patients for clozapine, haloperidol, lithium carbonate, pipamperone, risperidone, and valproate in patients ≥ 65 years. The median dosage was even lower in ADR patients treated with amisulpride, pregabalin, and quetiapine compared to all patients exposed in this age group. Among patients < 65 years in ADR cases involving amisulpride and amitriptyline, the dosages administered were higher compared to all patients exposed to the respective drug, and lower in ADR cases imputing citalopram, clozapine, escitalopram, and pregabalin.

**Table 4:** Median daily dosages in all patients exposed compared to patients with ADR cases under treatment with imputed drugs

Drug	Patients ≥ 65 years of age		Patients < 65 years of age	
	Median dose in mg/d (Min./Max.), all patients exposed	Median dose in mg/d (Min./Max.), ADR cases	Median dose in mg/d (Min./Max.), all patients exposed	Median dose in mg/d (Min./Max.), ADR cases
Amisulpride	300 (50/1600)	275 (50/1000)	500 (25/2000)	600 (100/1200)
Amitriptyline	75 (10/300)	75 (25/250)	100 (10/350)	125 (25/225)
Carbamazepine	400 (30/1800)	400 (120/800)	600 (0.5/2000)	600 (100/3200)
Citalopram	20 (5/250)	20 (10/60)	30 (1/1000)	20 (3/60)
Clozapine	125 (2.40/950)	150 (6.25/700)	300 (2/1500)	250 (12.5/1000)
Escitalopram	10 (2.5/200)	10 (5/20)	15 (2/200)	10 (5/30)
Haloperidol	3 (0.1/65)	5 (1/15)	10 (0.45/130)	10 (2/40)
Lithium carbonate	450 (0.5/1800)	737.5 (225/1125)	900 (0.5/2700)	900 (450/1575)
Mirtazapine	30 (1/300)	30 (7.5/90)	30 (1/300)	30 (7.5/90)
Olanzapine	10 (1.25/50)	10 (2.5/40)	15 (0.5/70)	15 (2.5/60)
Pipamperone	40 (0.07/360)	80 (20/160)	40 (0.07/600)	40 (20/320)
Pregabalin	150 (2.5/625)	125 (25/450)	225 (1/1200)	187.5 (25/600)
Quetiapine	100 (1/1200)	75 (12.5/600)	300 (6.3/2400)	300 (23.08/1500)
Risperidone	1.5 (0.03/15)	2 (0.5/8)	4 (0.25/100)	4 (0.5/12)
Sertraline	75 (10/300)	75 (25/200)	100 (10/1150)	100 (25/200)
Valproate	600 (50/4000)	900 (150/3000)	1200 (1/6800)	1200 (150/6000)
Venlafaxine	150 (7.5/750)	150 (37.5/375)	150 (1/600)	150 (8/450)

*ADR* adverse drug reaction; *d* day; *min*. minimum; *max*. maximum

^*^Only drugs with at least 10 or more ADR cases (imputed alone)

### Course, countermeasures, and risk factors of ADRs

Table 5 shows the course, countermeasures, and risk factors for ADRs in both age groups. We observed a total of 19 ADRs (1.6% of 1212) which resulted in death among patients ≥ 65 years of age versus only 7 fatal ADRs (0.2% of 4517) among younger patients. In so, the RR for fatal ADRs was 6.39 times higher (CI 3.03–13.47; data not shown) in older patients. However, ADRs in older patients were also more likely to be completely resolved by the end of the observation period compared to younger patients (81.9% vs. 72.1%; *p* < 0.001).

**Table 5:** Course, countermeasures, and risk factors of adverse drug reactions according to age group (≥ 65 years and < 65 years)

	Patients ≥ 65 years of age		Patients < 65 years of age		Chi^2^-Test	
	N cases	% of ADRs (N = 1212)	N cases	% of ADRs (N = 4517)	*p*	χ^2^
**Severity/course of the ADR**						
Prolongation of inpatient stay	541	44.6	1862	41.2	0.032	4.576
Life threatening	37	3.1	109	2.4	0.274	1.196
Fatal outcome	19	1.6	7	0.2	< 0.001	42.213
Full recovery by end of observation period	992	81.9	3258	72.1	< 0.001	47.148
Improvement by end of observation period	151	12.5	736	16.3	0.001	10.742
Unchanged by end of observation period	50	4.1	516	11.4	< 0.001	44.490
Countermeasures*						
None	7	0.6	129	2.9	< 0.001	21.403
Reduction of dose	207	17.1	824	18.2	0.350	0.876
Discontinuation of drug	1049	86.6	3516	77.8	< 0.001	44.798
Transfer to different ward/hospital	170	14.0	360	8.0	< 0.001	41.752
Symptomatic treatment with drugs	457	37.7	1564	34.6	0.046	3.974
Non-pharmacological treatment of symptoms	245	20.2	793	17.6	0.033	4.553
Risk factors for ADRs*						
None	494	40.7	2791	61.8	< 0.001	172.767
Risk factors present	718	59.2	1726	38.2	< 0.001	172.767
Susceptibility for ADRs	79	6.5	326	7.2	0.429	0.626
Pre-existing organ damage	526	43.4	755	16.7	< 0.001	391.061

*ADR* adverse drug reaction; *N* number (of)

^*^More than one item may apply

The majority of ADRs received some form of treatment. Cases in which no countermeasures were taken were less common in older patients (0.6% vs. 2.9%; *p* < 0.001). The most common countermeasure was discontinuation of the implicated drug(s), which was performed in 86.6% of patients aged ≥ 65 years of age and 77.8% of patients aged < 65 years of age (*p* < 0.001). Older patients were significantly more likely to require transfer to a different ward for more specialized care (14.0% vs. 8.0%; *p* < 0.001).

Risk factors were identified more frequently in older than in younger patients (59.2% vs. 38.2%; *p* < 0.001). The most common risk factor was pre-existing organ damage, which was present in 43.4% of ADRs in older patients and only in 16.7% of ADRs in younger patients (*p* < 0.001).

### ADR cases with fatal outcomes

Table 6 gives an overview of the 26 ADRs with fatal outcomes observed in this study. The most common cause of death was bolus death in 5 cases (19.2% of the 26 fatal ADRs), 2 of which affected older patients. EPS, including one malignant neuroleptic syndrome (MNS) and 4 cases of severe parkinsonism, ended fatally in another 5 cases (19.2% of fatal ADRs), with 4 cases affecting older adults. In 3 cases (11.5% of fatal ADRs), all of which affected older adults, cardiovascular ADRs (severe hypotension in all 3 cases) resulted in death. Also, ileus and respiratoy insufficiency led to death in 3 cases, of which 2 fatal courses affected older patients for both ADRs. Fatal cases of agranulocytosis were observed 3 times (11.5% of fatal ADRs) as well, but only among patients ≥ 65 years of age.

**Table 6:** Adverse drug reactions with fatal outcomes according to age (≥ 65 vs < 65 years of age) including imputed psychotropic drugs

Average age (mean ± SD) Type of ADR	Patients ≥ 65 years of age		Patients < 65 years of age	
	69.2 ± 15.4 years		47.7 ± 10.9 years	
	N cases	Drugs imputed	N cases	Drugs imputed
Bolus death	2	Haloperidol	3	Haloperidol decanoate Haloperidol Chlorprothixene
				Promethazine Diazepam Benperidol
		Fluphenazine		Olanzapine Mianserin Lorazepam Darifenacin
(Sub)Ileus	2	Clozapine Venlafaxine Duspatalin	1	Clozapine Pirenzepine
		Clozapine Perazine Pirenzepine Biperiden Amitriptyline Haloperidol Levomepromazine Chlorprothixene		
Sedation	1	Haloperidol Prothipendyl Mirtazapine	–	–
EPS (1 × MNS, 4 × severe parkinsonism)	4	Haloperidol Bupropion Lorazepam Diazepam	1	Fluphenazine Flupentixol Benperidol Diazepam
		Olanzapine Prothipendyl Haloperidol		
		Risperidone		
		Haloperidol decanoate Benperidol		
Seizure	1	Olanzapine Venlafaxine Prednisolon Methotrexate Fesoterodine	–	–
Respiratory insufficiency/arrest, pneumonia	2	Risperidone Mirtazapine Prothipendyl Melperone Tilidine	1	Haloperidol Levomepromazine
		Haloperidol Diazepam		
Cardiovascular ADRs (3 × severe hypotension	3	Tranylcypromine Bromazepam Prothipendyl Melperone Felodipin Bisoprolol Isosorbide dinitrate Hydrochlorothiazide Morphine sulfate	–	–
		Nortriptyline		
		Haloperidol Prothipendyl Melperone Metoprolol Enalapril Torasemide		
Agranulocytosis	3	Aripiprazole Enoxaparin	–	–
		Clozapine Quetiapine		
Gastrointestinal bleeding	1	Citalopram Acetylsalicylic acid	–	–
Suicide following prolonged impotence	–	–	1	Haloperidol Haloperidol decanoate Risperidone
Total number of cases	19		7	

*ADR* adverse drug reaction; *SD* standard deviation; *N* number (of); *EPS* extrapyramidal symptoms; *MNS* malignant neuroleptic syndrome

All cases of fatal ADRs in patients < 65 years of age imputed multiple drugs. High-potency FGAs were implicated in 4 cases, most often haloperidol (decanoate) in a total of 3 cases. SGAs, benzodiazepines and low potency FGAs were each imputed in 3 cases. An ADD (i.e., mianserin) was implicated in a single fatal ADR in this age group.

Among the 19 cases of fatal ADRs in patients ≥ 65 years of age, 4 cases implicated a single drug. High potency FGAs were implicated in 9 cases (2 single implications), whereby haloperidol (decanoate) was the most commonly imputed drug (8 cases; 1 single imputation in a patient treated with haloperidol per os). SGAs were implicated in 8 cases (1 single imputation), most commonly clozapine (4 cases) and olanzapine (2 cases). Risperidone was implicated in 2 ADRs (1 single imputation). ADDs were implicated in 9 cases (1 single imputation), most often mirtazapine (3 cases); TCAs and venlafaxine were imputed in 2 cases each, 1 single imputation with a TCA.

## Discussion

The present study analyzed the risk of ADRs within the inpatient psychiatric setting according to age (i.e., < 65 and ≥ 65 years of age). While the overall risk for ADRs did not differ between the two age groups, older patients were at higher risk for certain ADRs such as delirium, ataxia, certain types of EPS (e.g., parkinsonism, Pisa-/metronome syndrome), cardiovascular symptoms, and falls. Other ADRs such as suicidality, acute dystonia, akathisia, liver dysfunction, weight gain, sexual dysfunction, and hyperprolactinemia/galactorrhea were more common in younger patients. Older patients treated with ADDs—especially SSRIs and SNRIs—, low potency FGAs, and lithium had a higher risk of ADRs than younger patients, while younger patients treated with SGAs had a higher risk of ADRs than older patients treated with these drugs. Further, we found that ADRs in older patients were more likely to involve multiple drugs.

Older age is a well-described risk factor for ADRs [23]. While risk factors, such as pre-existing organ damage, are more common in patients ≥ 65 years of age, as found in this study, we were unable to detect a higher rate of ADRs in older adults. However, in this study, ADRs in older patients were more likely to be classified as life-threatening and required specialized care (Table 5). In addition, we found that older patients who experienced ADRs had an 6.4-fold increased risk of a fatal outcome. This significantly higher mortality greatly exceeds the findings of Dubrall et al., who examined ADRs reported to the German Federal Institute for Drugs and Medical Devices and found that ADR-related mortality was 3 times higher among patients ≥ 65 years of age than in those < 65 years of age [24]. The inpatient setting of our study, suggesting patients are more severely ill, may be one reason for this.

Moreover, we found that ADRs were more likely to affect patients with certain diagnoses. Younger patients with schizophrenia or acute mania had a significantly higher risk for ADRs than older patients with these diagnoses (Table 2). An explanation for this may be in the way these patients are treated: Kleimann et al. previously described remarkably high rates of polypsychopharmacy—defined as the intake of ≥ 4 psychotropic drugs—in patients with acute mania, which declined with higher age [25]. Further, Zolk et al. found that older schizophrenic patients were generally treated with lower doses of APDs [26]. Both aspects (i.e., lower doses, less polypsychopharmacy) may in turn reduce the risk of ADRs in these two diagnostic subgroups.

We found lower median doses in older patients for all drugs with the exception of venlafaxine (no difference between age groups or between patients with and without ADRs), sertraline (lowest dose in older patients with ADRs), and pipamperone (highest median dose in older patients with ADRs; Table 4). Compared to other ADDs, venlafaxine has well-characterized dose-dependent efficacy. Increasing venlafaxine dose to 150 mg yields benefits [27], which appears to be the target dose in both age groups. However, this seems to come at the expense of tolerability, as ADRs are apparently to be expected at this dose. Pipamerone, on the other hand, is voluptously used in geriatric patients [28] presumingly under the assumption that its use for these patients—even at high doses—is safe. Our data suggest, that this is not the case and higher pipamerone doses increase the risk for subsequent ADRs.

In general, we found a higher ADR risk in older patients treated with low potency ADRs than in younger patients (Fig. 3A, Table 3), especially prothipendyl, melperone, chlorprothixene, levomepromazine, and promethazine (Fig. 3B, Table 3). While promethazine’s, chlorprothixene’s, and levomepromazine’s anticholinergic effects (see below) surely contribute to this, melperone and prothipendyl lack this specific effect. Prothipendyl, a frequently used drug in Austrian nursing homes despite its classification as a potentially inappropriate drug, is not available in most European countries to due a higher risk of EPS [4, 29, 30]. A previous AMSP study additionally found a higher risk of cardiovascular ADRs under treatment with prothipendyl, even as a single imputation ADR [31]. Most ADRs imputed a low-potency FGA alongside other drugs, suggesting that pharmacokinetic and additive pharmacodynamic effects are the leading cause for ADRs in this drug group, while the capacity of low-potency FGAs to induce ADRs on their own was relatively low (suppl. Table 6). However, bearing in mind that this study presents relative risks, the absolute risk of ADRs unter treatment with most low-potency FGAs is comparable to or even lower than the ADR risk of other drugs with sedating properties, such as mirtazapine, olanzapine, and trazodone (Fig. 3B, suppl. Table 5).

In older adults, ADRs often present as nonspecific geriatric syndromes such as falls, delirium [32], decreased mobility, cognitive decline [33], and incontinence [12] possibly making them more difficult to accurately identify as drug-induced phenomena [32, 33]. Additionally, cognitive impairment may reduce the patient’s ability to adequately express any drug-related discomfort [34], perhaps explaining why dementia has even been found to decrease the risk of ADRs [13]. This is likely to have significantly contributed to the under-reporting of ADRs in cognitively impaired patients in the present study and emphasizes the importance of careful clinical monitoring, the collection of baseline parameters, and the explicit assessment of drug-related symptoms. Moreover, comorbidities may mask ADRs or be sufficient in themselves to explain a particular symptom. Additionally, certain symptoms, such as severe edema, may appear more alarming, when they occur in younger patients because they are unusual for this age group and are more likely to lack an alternate explanation other than drug-induced. Finally, older patients with conditions such as schizophrenia have most likely been treated with psychotropic drugs for an extended period of time, reducing the likelihood of ADRs that generally emerge early in treatment, whereas long-standing ADRs become more challenging to recognize.

In the present study, we identified age-related differences in the risk of various ADRs. A selection of findings will be discussed in detail below.

### Delirium and central anticholinergic effects

Unsurprisingly, we found that older patients had a higher risk of drug-induced delirium (Fig. 1B, Table 3), consistent with the observations reported by Greil et al. [14]. Up to 39% of deliriums in older hospitalized patients are associated with drug use. One of the most concerning drug properties in this regard is a high affinity for antimuscarinic acetylcholine receptors [35]. Central anticholinergic properties may be particularly harmful in older adults, which is why drugs such as amitriptyline, biperiden, and olanzapine are not generally recommended for older patients [4, 5], especially when multiple drugs with anticholinergic properties are combined [34]. Using AMSP data, Friedrich et al. previously found that APDs and ADDs with potent anticholinergic properties, such as TCAs and several SGAs (e.g., clozapine, olanzapine), have a higher propensity to cause drug-induced delirium in psychiatric inpatients. Clozapine and amitriptyline were the psychotropic drugs most frequently associated with drug-induced delirium, and most cases of drug-induced delirium were caused by multiple drugs [18]. In the present study, older patients treated with several drugs with strong anticholinergic properties, such as amitriptyline, trimipramine, levomepromazine, promethazine, chlorprothixene, and biperiden, did indeed have a significantly higher risk of experiencing ADRs than younger patients (Fig. 2B, suppl. Table 5). In both age groups, drug-induced delirium in the present study was often the effect of multiple drugs, indicating that this ADR typically results from pharmacodynamic drug-drug interactions. However, older patients were also at higher risk of experiencing delirium imputing a single drug (Fig. 2A, suppl. Table 6 and 7).

### Suicidality and serotonergic ADRs

Early warnings by the US Food and Drug Administration in 2004 highlighted the risk of suicidality associated with the use of SSRIs in children, adolescents, and young adults under 25, primarily during the initial stages of treatment [36]. While this rare effect has also been noted in patients above the age of 25 [37, 38], the risk significantly declines with age [37]. This is consistent with our findings indicating only a single instance of this ADR in older patients (Table 3). Although the exact mechanisms of drug-induced suicidality remain elusive, serotonergic activation induced by SSRIs and SNRIs is proposed to significantly contribute [39]. Notably, our study found older patients had an overall higher risk for serotonergic ADRs (including serotonin syndrome; Fig. 1B, Table 3), especially as a multiple imputation ADR (Fig. 2B, suppl. Table 4). Serotonin syndrome, though rare, preferentially affects high-risk patients, i.e., critically ill patients and those with polypharmacy, and often goes unrecognized [40].

### Extrapyramidal symptoms

Affecting about 1 in 5 patients [41], EPS are one of the most significant ADRs of treatment with APDs and a major concern in older patients [4, 13]. When systematically analyzed, drug-induced movement disorders occur more frequently in older adults [42]. However, distinguishing new-onset drug-induced EPS from pre-existing movement disorders may pose a challenge [42], increasing the risk that they remain unnoticed [43]. This may in part explain why the overall incidence of EPS in this study did not differ between older and younger patients (Fig. 1A, Table 3). The study by Greil et al. using Swiss AMSP data indicated that the risk for EPS decreases with age, though with limited statistical significance (*p* < 0.05) [14]. However, in examining a much larger patient collective, we found that individual types of EPS showed age-related effects, which was not previously considered by Greil et al. [14]. The risk for some types of movement disorders, such as acute dystonia and akathisia, was higher in younger patients in the present study (Fig. 1B, Table 3). Indeed, apart from male sex, younger age is a well-known risk factor for acute dystonia [44]. The likelihood of developing akathisia and acute dystonia increases with the use of high doses and rapid titration strategies and is highest when antipsychotic treatment is first initiated, all of which may affect younger patients more often [44, 45].

On the other hand, this study found that the risk for other types of EPS was higher in older patients (i.e., parkinsonism, Pisa/metronome syndrome; Fig. 1B, Table 3) and EPS among older patients were more likely to result in death (4 out of 5 fatal EPS cases; Table 6). Parkinsonism occurs in up to 50% of older patients treated with APDs and up to 67% of those with dementia [46] and often occurs even when APDs are used at lower than usual doses [42]. An earlier analysis of severe parkinsonism using AMSP data found that pre-existing organic brain damage (such as dementia) is a relevant risk factor for APD-induced parkinsonism and that high-potency FGAs expectably have an expectably higher risk than SGAs or low-potency FGAs [47]. Further, both older age and organic brain damage are known risk factors for Pisa syndrome and the related condition, metronome syndrome, which can emerge either acutely or after prolonged exposure to APDs [48, 49].

### Seizures

In the present study, younger patients had a significantly higher risk for drug-induced seizures than older patients (Fig. 1B, Table 3). Druschky et al., who analyzed the occurrence of APD-induced seizures within the AMSP database over a slightly shorter time period (i.e., 1993 to 2015) found that young men with schizophrenia were most at risk for this ADR. The by far highest risk of seizures was found for clozapine, with a comparatively low rate for risperidone [50], an APD with high use among older patients [26]. ADD-associated seizures are rare, but seem to particulary be associated with the use of TCAs and tend to affect younger men and patients suffering from schizophrenia [51]. While certain risk factors for drug-induced seizures, such as higher doses [52], are presumably more prevalent in younger patients, other significant risk factors, such as somatic comorbidities [53], pre-existing brain damage, and EEG abnormalities [52], are more common in older adults. But again, the co-occurrence of these risk factors may make it more difficult to definitively attribute a seizure to drug use, resulting in an only “possible” probability rating for the involvement of a psychotropic drug among older patients.

### Liver dysfunction

Our results suggest that the risk for elevated transaminases is higher in younger patients (Fig. 1). Liver injury associated with APDs is most often associated with olanzapine, followed by perazine and clozapine [16], therefore providing one explanation for the overall higher risk of ADRs under olanzapine and clozapine in younger patients (Fig. 2A, suppl. Table 5). Drug-induced liver injury caused by ADDs most commonly implicate mianserine and agomelatine [54]. Greil et al. also suggested a higher risk of psychotropic-drug induced liver dysfunction in younger patients, though the effect was statistically weak (*p* < 0.05) [14]. Whether age is a susceptibility factor for drug-induced liver injury appears to be drug-specific. However, persistent liver injury is appears to be more common with higher age [55].

### Sexual dysfunction, galactorrhea/hyperprolactinemia, and weight gain

The present study found only one case of an ADR presenting with genital dysfunction in older patients, making it one of the main ADRs that are significantly more common in younger patients (Fig. 1A, Table 3). In general, the prevalence of sexual dysfunction increases with age [56] and in the presence of comorbidities such as hypertension, diabetes, and benign prostatic hyperplasia [57], making it more difficult to identify drug-induced effects. Moreover, this often shame-filled ADR is significantly under-reported [58], especially among older patients [59].

Similarly, symptomatic prolactin elevation and events of galactorrhea were significantly less common in older patients (Fig. 1B, Table 3), as was also found in the earlier publication of Greil et al. [14]. Amenorrhea, a possible symptom of hyperprolactinemia, is expected to occur only in premenopausal women, but breast tissue growth, galactorrhea, or sexual dysfunction may still affect older adults [60]. The clinical implications of elevated prolactin in older adults may also be less apparent. For example, (chronic) hyperprolactinemia is associated with osteoporosis [61] and certain types of breast cancer [62], both of which are complex conditions difficult to causally attribute to drug use. Previous studies suggest the prevalence of hyperprolactinemia is indeed higher in premenopausal (53–65.6%) than postmenopausal women (32–45.1%) [63, 64]. However, the risk detected in the present study is significantly lower, as only severe case with acute symptoms are included. Apart from amisulpride, risperidone has a particularly high propensity to cause hyperprolactinema [17]. Risperidone is also one of the most commonly used APDs in older patients [26], suggesting this ADR should be more common. However, the dose-dependency of hyperprolactinemia [65] may mitigante this effect, as older patients—even those with ADRs—were treated with lower median doses of risperidone (Table 4).

Weight gain is often a primary concern in patients treated with psychotropic drugs Consistent with the findings of Greil et al. [14] as well as other authors [66, 67], our study found that the risk for psychotropic drug-induced weight gain was significantly higher in younger patients (Fig. 1B, Table 3). Using AMSP data, Schneider et al. previously reported that olanzapine, quetiapine, risperidone, mirtazapine, and valproate were among the drugs most often associated with psychotropic drug-induced weight gain [15]. The higher propensity of these four drugs to cause this ADR may explain their higher ADR risk in younger patients in this study. Additionally, younger patients in the present study were treated with higher median doses of quetiapine, valproate, and risperidone, contributing to the risk of weight gain, which appears to have dose-dependent effects [68].

### Cardiovascular adverse reactions

Cardiovascular ADRs are a major concern in older patients. The relevance of this ADR type is underlined by the 3 fatal cases of cardiovascular ADRs among older patients detected in the present study (Table 6). Because of their affinity for α_1_-adrengic receptors, APDs can cause hypotension [69]. In fact, a recent meta-analysis found that APDs, along with α-blockers and sodium–glucose-cotransporter (SGLT)-2 inhibitors, were the most common drug classes associated with orthostatic hypotension [70]. The risk of hypotension further increases when psychotropic drugs are used in combination with antihypertensive drugs, such as diuretics or β-blockers, or other psychotropic drugs [31, 71, 72]. Our study found that cardiovascular ADRs in older adults often imputed multiple drugs, whereas cardiovascular ADRs in younger patients often imputed a single drug (Fig. 2A). This is most likely due to a lower utilization of antihypertensive drugs in younger patients.

### Hyponatremia

Among the ADRs examined in this study, hyponatremia was one of the ADRs with the highest risk (i.e., 3.7-fold) for older compared to younger patients (Fig. 1B, Table 3), also explaining the higher risk of ADRs in older patients treated with SSRIs, and even more so with SNRIs (Fig. 2A, suppl. Table 2). Using AMSP data, Seifert et al. previously described that older patients, particularly women ≥ 65 years of age treated with SNRIs and other potentially hyponatremia-inducing drugs, were the most vulnerable patient group for this ADR [19]. Among psychotropic drugs, SSRIs and SNRIs are best known for their propensity to cause this ADR, especially at the beginning of treatment and, therefore, even at lower doses [19]. This potentially explains the lowest sertraline dose in older patients with ADRs (Table 4). The risk for hyponatremia increases when SSRI and SNRI are used in combination with angiotensin-converting enzyme (ACE) inhibitors, thiazide and thiazide-like diuretics, and proton pump inhibitors [19, 73, 74], inducing additive pharmacodynamic effects [19, 75]. However, the risk for hyponatremia associated with single psychotropic drug, did not differ between the two age groups (Fig. 2B, suppl. Table 3).

### Falls

We found that older patients had a sixfold higher risk of falls as an ADR compared to younger patients (Fig. 1B, Table 3). As falls are a common occurrence with a 2-year prevalence of 36% of patients aged ≥ 65 years [76] and psychotropic drug use indisputably contributes to this risk [7], this was to be expected. It is difficult to determine which psychotropic drugs carry the greatest risk of falls. It appears, however, that long-acting benzodiazepines and SSRIs may pose a particular risk [7]. The present study found drug-induced falls, especially in older patients, imputed multiple drugs significantly more often than a single drug (Fig. 2B, suppl. Tables 3 and 4), suggesting that psychodynamic drug–drug interactions are a relevant contributor to this ADR.

## Strengths and limitations

AMSP is a structured pharmacovigilance program with an established methodology that assesses drug safety in the “real world” psychiatric inpatient setting. As clinical trials often exclude older patients and patients with polypharmacy, pharmacovigilance systems such as AMSP are indispensable for assessing ADRs in this population. Further, because data is collected in a uniform manner and ADRs are carefully analyzed by multiple drug safety expert teams prior to their inclusion in the AMSP database, AMSP has a high accuracy of correct causal attribution of drugs involved in the respected ADRs.

Nevertheless, the present study has several limitations that must be discussed. Firstly, AMSP is not an RCT, limiting the reliability of evidence. Several studies using AMSP data have highlighted changing drug utilization trends over time [77, 78], alongside regional drug utilization trends and regional drug availability. While hospitals from Germany and Switzerland contributed to AMSP as of 1993, Austria has been participating since 2001. Due to the database structure, it is not possible to distinguish whether a patient experienced multiple ADRs. Next to a detailed assessment of a patient’s drug use, the epidemiologic data on patients under surveillance gathered by AMSP only includes a limited amount of information (i.e., diagnoses, age, sex). Underreporting of ADRs is likely as physicians who serve as drug monitors generally do this alongside their clinical work. Therefore reporting of ADRs is subjective to their personal time, motivation, as well as the financial resources of the participating hospital. This may also contribute to an individual and/or institutional bias: ADRs occurring in patients treated with drugs better known for their potentially severe ADRs (e.g., TCAs for their delirogenic potential) may be more frequently detected and/or documented. ADRs may also be more difficult to detect in elderly patients, especially in those suffering from dementia due to their reduced ability to adequately report symptoms, resulting in falsely low ADR rates within this patient population.

## Conclusion and clinical implications

The present study indicates that the risk of several types of psychotropic drug-induced ADRs, such as hyponatremia, delirium, weight gain, sexual dysfunction, and galactorrhea, shows age-dependent effects. Drugs which are often considered relatively “harmless”, such as SSRI, SNRI, and low potency FGAs, are associated with a significantly higher risk of ADRs in older patients compared to younger patients. Clinicians should be aware of age-dependent risk factors for ADRs and proactively monitor patients, starting with a baseline assessment. Regularly including (clinical) pharmacists in the treatment of inpatients has proven a promising approach in reducing drug-related problems in mental health care [79]. Additionally, tools such as therapeutic drug monitoring (TDM) are invaluable in guiding appropriate dosing, especially in patients with somatic diseases, such as renal or hepatic failure, polypharmacy, or a history of ADRs, in order to lower the risk for (dose-dependent) ADRs. Pharmacogenetic testing may also present a unique opportunity to further individualize drug treatment, thus optimizing drug safety.

## Supplementary Information

Supplementary Table 1

Supplementary Table 2

Supplementary Table 3

Supplementary Table 4

Supplementary Table 5

Supplementary Table 6

Supplementary Table 7

## Data Availability

Data on an individual level generated and/or analysed during the current study are not publicly available due to data protection regulations and ethical considerations. Summarized data on the prevalence of ADRs and drug use relevant to the present study is provided in the tables and supplementary material of this manuscript.

## Abbreviations

ACE: Angiotensin-converting enzyme
ADD: Antidepressant drug
AED: Antiepileptic drug
ADR: Adverse drug reaction
AMSP: Drug Safety in Psychiatry (German: “Arzneimittelsicherheit in der Psychiatrie”)
AParkD: Antiparkinson drug
APD: Antipsychotic drug
CI: Confidence interval
EPS: Extrapyramidal symptoms
FGA: First-generation antipsychotic drug
hp: High potency
HYPD: Hypnotic drug
lp: Low potency
SGA: Second-generation antipsychotic drug
SNRI: Selective serotonin-norepinephrine reuptake inhibitor
SSRI: Selective serotonin reuptake inhibitor
TCA: Tricyclic antidepressant drug
TRD: Tranquilizing drug
w/: With
w/o: Without
NaSSA: Noradrenergic and specific serotonergic antidepressant
MNS: Malignant neuroleptic syndrome
N: Number (of)
d: Day
Min.: Mininmum
Max.: Maximum
df: Degrees of freedom
ICD-10: International Classification of Disease, 10th Version
