# Supplementary material for: Psychotropic drug-induced adverse drug reactions in 462,661 psychiatric inpatients in relation to age: results from a German drug surveillance program from 1993–2016
Source: Ann Gen Psychiatry. 2024 Nov 18;23:47. doi: 10.1186/s12991-024-00530-0 (PMC11575432; doi:10.1186/s12991-024-00530-0)
Supplement: Supplementary file 1 — Supplementary Table 1 [file 12991_2024_530_MOESM1_ESM.docx]

**Supplementary Table 1:** Classification of psychotropic drugs relevant to this study.

| **Psychotropic drug group** | | **Subgroup** | **Individual drugs^1^** |
| --- | --- | --- | --- |
| ADD | | *SSRI* | citalopram, escitalopram, paroxetine, sertraline |
|  |  | *SNRI* | duloxetine, venlafaxine |
|  |  | *TCA* | amitriptyline, clomipramine, doxepin, nortriptyline, trimipramine, |
|  |  | *NaSSA* | mirtazapine, mianserine |
|  |  | *other ADD* | agomelatine, bupropion, trazodone |
| APD | FGA | *lp FGA* | chlorprothixene, melperone, pipamperone, promethazine, prothipendyl |
|  |  | *hp FGA* | flupentixol, haloperidol, perazine |
|  | SGA |  | amisulpride, clozapine, olanzapine, quetiapine, risperidone, aripiprazole |
| HYPD | | *Z-drugs* | zolpidem, zopiclone |
|  |  | *others* | valerian |
| TRD | | *benzodiazepines* | alprazolam, diazepam, lorazepam, oxazepam |
| AED | |  | carbamazepine, lamotrigine, pregabalin, valproate |
| Antiparkinson drugs | |  | biperiden |

**ADD:** antidepressant drug; **SSRI:** selective serotonin reuptake inhibitor; **SNRI:** selective serotonin-norepinephrine reuptake inhibitor; **TCA:** tricyclic antidepressant; **NaSSA:** noradrenergic and specific serotonergic antidepressant; **APD:** antipsychotic drug; **FGA:** “first-generation” antipsychotic drug; **lp:** low potency; **hp:** high potency; **SGA:** “second-generation” antipsychotic drug; **HYPD:** hypnotic drug; **TRD:** tranquilizing drug; **AED:** antiepileptic drug

^1^ only drugs used in the treatment of ≥1000 patients ≥65 years are listed
