# Supplementary material for: Psychotropic drug-induced adverse drug reactions in 462,661 psychiatric inpatients in relation to age: results from a German drug surveillance program from 1993–2016
Source: Ann Gen Psychiatry. 2024 Nov 18;23:47. doi: 10.1186/s12991-024-00530-0 (PMC11575432; doi:10.1186/s12991-024-00530-0)
Supplement: Supplementary file 2 — Supplementary Table 2 [file 12991_2024_530_MOESM2_ESM.docx]

**Suppl. Table 2**: Number of different types of drugs in patients ≥ 65 vs. < 65 years (Table 2A: all patients, Table 2B: patients with adverse drug reactions).

**A:**

|  | **All patients ≥65 years of age (N=99,099)** | **All patients <65 years of age (N=363,562)** | **Unpaired t-test** | | |
| --- | --- | --- | --- | --- | --- |
|  | **Mean ± SD** | **Mean ± SD** | **t-statistic** | ***p*-value** | **Cohen's *d*** |
| **N drugs** | 5.37 ± 2.58 | 3.50 ± 2.10 | 76.060 | <0.001 | 0.845 |
| **N psychotropic drugs** | 2.62 ± 1.27 | 2.54 ± 1.32 | 6.183 | <0.001 | 0.061 |
| **N antipsychotic drugs** | 1.30 ± 0.53 | 1.44 ± 0.66 | -20.891 | <0.001 | -0.221 |
| **N antidepressant drugs** | 1.24 ± 0.46 | 1.23 ± 0.45 | 1.630 | 0.103 | 0.022 |

**B:**

|  | **Patients ≥65 years of age with ≥1 ADR (N=1212)** | **Patients <65 years of age with ≥1 ADR (N=4517)** | **Unpaired t-test** | | |
| --- | --- | --- | --- | --- | --- |
|  | **Mean ± SD** | **Mean ± SD** | **t-statistic** | ***p*-value** | **Cohen's *d*** |
| **N drugs** | 5.45 ± 2.59 | 3.49 ± 2.00 | 29.852 | <0.001 | 0.907 |
| **N psychotropic drugs** | 2.81 ± 1.30 | 2.70 ± 1.36 | 3.013 | 0.003 | 0.082 |
| **N antipsychotic drugs** | 1.50 ± 0.71 | 1.40 ± 0.64 | 4.460 | <0.001 | 0.153 |
| **N antidepressant drugs** | 1.27 ± 0.50 | 1.28 ± 0.49 | -0.551 | 0.581 | -0.020 |

**N:** number (of); **SD:** standard deviation; **ADR:** adverse drug reaction
