# Supplementary material for: Psychotropic drug-induced adverse drug reactions in 462,661 psychiatric inpatients in relation to age: results from a German drug surveillance program from 1993–2016
Source: Ann Gen Psychiatry. 2024 Nov 18;23:47. doi: 10.1186/s12991-024-00530-0 (PMC11575432; doi:10.1186/s12991-024-00530-0)
Supplement: Supplementary file 3 — Supplementary Table 3 [file 12991_2024_530_MOESM3_ESM.docx]

**Suppl. Table 3**: Incidence and relative risk of different types of adverse drug reactions (single imputations) in older vs. younger patients

| **Adverse drug reaction** | **Patients ≥65 years of age (N = 99,099)** | | **Patients <65 years of age (N = 363,562)** | | **Older vs. younger patients** |
| --- | --- | --- | --- | --- | --- |
|  | **N cases** | **% of patients**  **(95% CI)** | **N cases** | **% of patients**  **(95% CI)** | **RR (95% CI)** |
| **All ADRs*** | 698 | **0.704%** | 3212 | **0.883%** | **0.80 (0.73–0.87)** |
| **Delirium, confusio*** | 45 | **0.045%** | 59 | **0.016%** | **2.80 (1.90–4.12)** |
| Delirium* | 41 | **0.041%** | 53 | **0.015%** | **2.84 (1.89–4.27)** |
| **Psychiatric symptoms excl. delirium** | 60 | **0.061%** | 210 | **0.058%** | **1.05 (0.79–1.40)** |
| Disturbance of consciousness* | 8 | **0.008%** | 12 | **0.003%** | **2.45 (1.00–5.98)** |
| Psychosis/(pseudo-) hallucinations | 6 | **0.006%** | 35 | **0.010%** | **0.63 (0.26–1.50)** |
| Restlessness/agitation | 20 | **0.020%** | 60 | **0.017%** | **1.22 (0.74–2.03)** |
| Sedation | 9 | **0.009%** | 22 | **0.006%** | **1.50 (0.69–3.26)** |
| Suicidality | 1 | **0.001%** | 23 | **0.006%** | **0.16 (0.02–1.18)** |
| **Neurological symptoms excl. EPS** | 80 | **0.081%** | 326 | **0.090%** | **0.90 (0.70–1.15)** |
| Seizures | 8 | **0.008%** | 50 | **0.014%** | **0.59 (0.28–1.24)** |
| Myoclonus | 5 | **0.005%** | 17 | **0.005%** | **1.08 (0.40–2.92)** |
| Ataxia | 8 | **0.008%** | 13 | **0.004%** | **2.26 (0.94–5.45)** |
| Tremor | 12 | **0.012%** | 38 | **0.010%** | **1.16 (0.61–2.22)** |
| Vision disorders, glaucoma | 4 | **0.004%** | 25 | **0.007%** | **0.59 (0.20–1.69)** |
| Serotonin-syndrome, serotonergic ADRs | 9 | **0.009%** | 21 | **0.006%** | **1.57 (0.72–3.43)** |
| Restless legs/arms* | 6 | **0.006%** | 60 | **0.017%** | **0.37 (0.16–0.85)** |
| **EPS** | 128 | **0.129%** | 465 | **0.128%** | **1.01 (0.83–1.23)** |
| Neuroleptic malignant syndrome | 4 | **0.004%** | 19 | **0.005%** | **0.77 (0.26–2.27)** |
| Tardive dyskinesia | 6 | **0.006%** | 29 | **0.008%** | **0.76 (0.32–1.83)** |
| Pisa/metronome-syndrome* | 27 | **0.027%** | 19 | **0.005%** | **5.21 (2.90–9.38)** |
| Atypical dyskinesia | 9 | **0.009%** | 37 | **0.010%** | **0.89 (0.43–1.85)** |
| Acute dystonia* | 5 | **0.005%** | 135 | **0.037%** | **0.14 (0.06–0.33)** |
| Parkinsonism | 53 | **0.053%** | 97 | **0.027%** | **2.00 (1.43–2.80)** |
| Akathisia* | 14 | **0.014%** | 120 | **0.033%** | **0.43 (0.25–0.74)** |
| **Gastrointestinal disorders** | 42 | **0.042%** | 127 | **0.035%** | **1.21 (0.86–1.72)** |
| (Sub)ileus/severe constipation | 5 | **0.005%** | 15 | **0.004%** | **1.22 (0.44–3.36)** |
| Nausea/vomiting | 12 | **0.012%** | 35 | **0.010%** | **1.26 (0.65–2.42)** |
| **Liver dysfunction*** | 41 | **0.041%** | 274 | **0.075%** | **0.55 (0.40–0.76)** |
| Elevated transaminases* | 41 | **0.041%** | 274 | **0.075%** | **0.55 (0.40–0.76)** |
| **Cutaneous reactions** | 130 | **0.131%** | 513 | **0.141%** | **0.93 (0.77–1.13)** |
| Edema | 43 | **0.043%** | 158 | **0.043%** | **1.00 (0.71–1.40)** |
| Allergic cutaneous reactions | 78 | **0.079%** | 313 | **0.086%** | **0.91 (0.71–1.17)** |
| **Cardiovascular disorders** | 61 | **0.062%** | 176 | **0.048%** | **1.27 (0.95–1.70)** |
| (Orthostatic) syncope* | 25 | **0.025%** | 50 | **0.014%** | **1.83 (1.13–2.96)** |
| Symptomatic hypotension w/ vertigo | 5 | **0.005%** | 18 | **0.005%** | **1.02 (0.38–2.74)** |
| Arrhythmia | 13 | **0.013%** | 51 | **0.014%** | **0.94 (0.51–1.72)** |
| Prolonged QT-interval | 6 | **0.006%** | 20 | **0.006%** | **1.10 (0.44–2.74)** |
| **Urological dysfunction** | 14 | **0.014%** | 56 | **0.015%** | **0.92 (0.51–1.65)** |
| Urinary retention | 8 | **0.008%** | 25 | **0.007%** | **1.17 (0.53–2.60)** |
| **Genital dysfunction*** | 1 | **0.001%** | 89 | **0.024%** | **0.04 (0.01–0.30)** |
| Erectile dysfunction | 0 | **0.000%** | 42 | **0.012%** | **–** |
| **Hematologic disorders** | 40 | **0.040%** | 180 | **0.050%** | **0.82 (0.58–1.15)** |
| Agranulocytosis | 9 | **0.009%** | 38 | **0.010%** | **0.87 (0.42–1.80)** |
| Neutropenia | 14 | **0.014%** | 76 | **0.021%** | **0.68 (0.38–1.20)** |
| Thrombocytopenia | 8 | **0.008%** | 24 | **0.007%** | **1.22 (0.55–2.72)** |
| **Metabolic disorders, electrolyte imbalances*** | 21 | **0.021%** | 257 | **0.071%** | **0.30 (0.19–0.47)** |
| Hyponatremia | 13 | **0.013%** | 48 | **0.013%** | **0.99 (0.54–1.83)** |
| Increased prolactin/galactorrhea* | 2 | **0.002%** | 163 | **0.045%** | **0.05 (0.01–0.18)** |
| **Changes in body weight*** | 8 | **0.008%** | 340 | **0.094%** | **0.09 (0.04–0.17)** |
| Weight gain* | 7 | **0.007%** | 340 | **0.094%** | **0.08 (0.04–0.16)** |
| **Others** | 30 | **0.030%** | 140 | **0.039%** | **0.79 (0.53–1.17)** |
| Falls | 3 | **0.003%** | 4 | **0.001%** | **2.75 (0.62–12.29)** |

*indicates a significant result

**N:** number (of); **RR:** relative risk; **CI:** confidence interval; **ADR**: adverse drug reaction; **EPS**: extrapyramidal symptoms
