# Supplementary material for: Psychotropic drug-induced adverse drug reactions in 462,661 psychiatric inpatients in relation to age: results from a German drug surveillance program from 1993–2016
Source: Ann Gen Psychiatry. 2024 Nov 18;23:47. doi: 10.1186/s12991-024-00530-0 (PMC11575432; doi:10.1186/s12991-024-00530-0)
Supplement: Supplementary file 4 — Supplementary Table 4 [file 12991_2024_530_MOESM4_ESM.docx]

**Suppl. Table 4**: Incidence and relative risk of different types of adverse drug reactions (multiple imputations) in older vs. younger patients

| **Adverse drug reaction** | **Patients ≥65 years of age (N = 99,099)** | | **Patients <65 years of age (N = 363,562)** | | **Older vs. younger patients** |
| --- | --- | --- | --- | --- | --- |
|  | **N cases** | **% of patients**  **(95% CI)** | **N cases** | **% of patients**  **(95% CI)** | **RR (95% CI)** |
| **All ADRs*** | 514 | **0.519%** | 1305 | **0.359%** | **1.44 (1.30–1.60)** |
| **Delirium, confusion*** | 73 | **0.074%** | 125 | **0.034%** | **2.14 (1.61–2.86)** |
| Delirium* | 68 | **0.069%** | 117 | **0.032%** | **2.13 (1.58–2.88)** |
| **Psychiatric symptoms excl. Delirium*** | 27 | **0.027%** | 60 | **0.017%** | **1.65 (1.05–2.60)** |
| Disturbance of consciousness | 12 | **0.012%** | 22 | **0.006%** | **2.00 (0.99–4.04)** |
| Psychosis/(pseudo-) hallucinations | 3 | **0.003%** | 4 | **0.001%** | **2.75 (0.62–12.29)** |
| Restlessness/agitation | 4 | **0.004%** | 6 | **0.002%** | **2.45 (0.69–8.67)** |
| Sedation | 7 | **0.007%** | 15 | **0.004%** | **1.71 (0.70–4.20)** |
| Suicidality | 0 | **0.000%** | 11 | **0.003%** | **–** |
| **Neurological symptoms excl. EPS** | 52 | **0.052%** | 207 | **0.057%** | **0.92 (0.68–1.25)** |
| Seizures* | 16 | **0.016%** | 112 | **0.031%** | **0.52 (0.31–0.88)** |
| Myoclonus | 1 | **0.001%** | 18 | **0.005%** | **0.20 (0.03–1.53)** |
| Ataxia* | 12 | **0.012%** | 11 | **0.003%** | **4.00 (1.77–9.07)** |
| Tremor | 8 | **0.008%** | 24 | **0.007%** | **1.22 (0.55–2.72)** |
| Vision disorders, glaucoma | 1 | **0.001%** | 5 | **0.001%** | **0.73 (0.09–6.28)** |
| Serotonin-syndrome, serotonergic ADRs* | 10 | **0.010%** | 14 | **0.004%** | **2.62 (1.16–5.90)** |
| Restless legs/arms | 0 | **0.000%** | 3 | **0.001%** | **–** |
| **EPS** | 84 | **0.085%** | 247 | **0.068%** | **1.25 (0.97–1.60)** |
| Neuroleptic malignant syndrome | 7 | **0.007%** | 23 | **0.006%** | **1.12 (0.48–2.60)** |
| Tardive dyskinesia | 2 | **0.002%** | 16 | **0.004%** | **0.46 (0.11–1.99)** |
| Pisa/metronome-syndrome* | 30 | **0.030%** | 40 | **0.011%** | **2.75 (1.71–4.42)** |
| Atypical dyskinesia | 6 | **0.006%** | 18 | **0.005%** | **1.22 (0.49–3.08)** |
| Acute dystonia* | 5 | **0.005%** | 50 | **0.014%** | **0.37 (0.15–0.92)** |
| Parkinsonism* | 27 | **0.027%** | 58 | **0.016%** | **1.71 (1.08–2.70)** |
| Akathisia | 5 | **0.005%** | 27 | **0.007%** | **0.68 (0.26–1.76)** |
| **Gastrointestinal disorders** | 12 | **0.012%** | 35 | **0.010%** | **1.26 (0.65–2.42)** |
| (Sub)ileus/severe constipation | 6 | **0.006%** | 23 | **0.006%** | **0.96 (0.39–2.35)** |
| Nausea/vomiting | 1 | **0.001%** | 1 | **0.000%** | **3.67 (0.23–58.66)** |
| **Liver dysfunction** | 20 | **0.020%** | 84 | **0.023%** | **0.87 (0.54–1.42)** |
| Elevated transaminases | 20 | **0.020%** | 82 | **0.023%** | **0.89 (0.55–1.46)** |
| **Cutaneous reactions** | 7 | **0.007%** | 28 | **0.008%** | **0.92 (0.40–2.10)** |
| Edema | 6 | **0.006%** | 24 | **0.007%** | **0.92 (0.37–2.24)** |
| Allergic cutaneous reactions | 2 | **0.002%** | 0 | **0.000%** | **–** |
| **Cardiovascular disorders*** | 74 | **0.075%** | 94 | **0.026%** | **2.89 (2.13–3.92)** |
| (Orthostatic) syncope* | 37 | **0.037%** | 46 | **0.013%** | **2.95 (1.91–4.55)** |
| Symptomatic hypotension w/ vertigo* | 18 | **0.018%** | 8 | **0.002%** | **8.25 (3.59–18.98)** |
| Arrhythmia | 11 | **0.011%** | 26 | **0.007%** | **1.55 (0.77–3.14)** |
| Prolonged QT-interval | 4 | **0.004%** | 12 | **0.003%** | **1.22 (0.39–3.79)** |
| **Urological dysfunction** | 21 | **0.021%** | 84 | **0.023%** | **0.92 (0.57–1.48)** |
| Urinary retention | 19 | **0.019%** | 72 | **0.020%** | **0.97 (0.58–1.61)** |
| **Genital dysfunction** | 0 | **0.000%** | 17 | **0.005%** | **–** |
| Erectile dysfunction | 0 | **0.000%** | 23 | **0.006%** | **–** |
| **Hematologic disorders*** | 16 | **0.016%** | 29 | **0.008%** | **2.02 (1.10–3.73)** |
| Agranulocytosis | 3 | **0.003%** | 5 | **0.001%** | **2.20 (0.53–9.21)** |
| Neutropenia | 4 | **0.004%** | 13 | **0.004%** | **1.13 (0.37–3.46)** |
| Thrombocytopenia | 1 | **0.001%** | 6 | **0.002%** | **0.61 (0.07–5.08)** |
| **Metabolic disorders, electrolyte imbalances*** | 93 | **0.094%** | 107 | **0.029%** | **3.19 (2.41–4.21)** |
| Hyponatremia* | 93 | **0.094%** | 56 | **0.015%** | **6.09 (4.37–8.49)** |
| Increased prolactin/galactorrhea* | 1 | **0.001%** | 43 | **0.012%** | **0.09 (0.01–0.62)** |
| **Changes in body weight*** | 4 | **0.004%** | 154 | **0.042%** | **0.10 (0.04–0.26)** |
| Weight gain* | 3 | **0.003%** | 153 | **0.042%** | **0.07 (0.02–0.23)** |
| **Others*** | 28 | **0.028%** | 34 | **0.009%** | **3.02 (1.83–4.98)** |
| Falls* | 21 | **0.021%** | 11 | **0.003%** | **7.00 (3.38–14.53)** |

*indicates a significant result

**N:** number (of); **RR:** relative risk; **CI:** confidence interval; **ADR**: adverse drug reaction; **EPS**: extrapyramidal symptoms
