# Supplementary material for: Psychotropic drug-induced adverse drug reactions in 462,661 psychiatric inpatients in relation to age: results from a German drug surveillance program from 1993–2016
Source: Ann Gen Psychiatry. 2024 Nov 18;23:47. doi: 10.1186/s12991-024-00530-0 (PMC11575432; doi:10.1186/s12991-024-00530-0)
Supplement: Supplementary file 5 — Supplementary Table 5 [file 12991_2024_530_MOESM5_ESM.docx]

**Suppl. Table 5:** Relative risk (including 95% confidence interval) and incidence (in % of patients exposed to the respective drug/drug group of adverse drug reactions (all imputations) of psychotropic drugs and drug groups according to age (≥ 65 and < 65 years)

| **Drug/drug group** | **Patients ≥65 years of age** | | | **Patients <65 years of age** | | | **Relative Risk (older vs. younger patients)** | | |
| --- | --- | --- | --- | --- | --- | --- | --- | --- | --- |
|  | **N cases of ADRs and number of patients exposed to respective drug (group)** | **N patients exposed to respective drug (group)** | **% of patients exposed to drug (group) with ADR** | **N cases of ADRs and number of patients exposed to respective drug (group)** | **N patients exposed to respective drug (group)** | **% of patients exposed to drug (group) with ADR** | **RR** | **LL** | **UL** |
| **Any psychotropic drug** | 1212 | 99,099 | 1.223% | 4517 | 363,562 | 1.242% | 0.98 | 0.95 | 1.02 |
| **Antidepressant drugs*** | 538 | 56,578 | 0.951% | 1341 | 187,010 | 0.717% | 1.33 | 1.26 | 1.40 |
| **SSRI*** | 153 | 19,333 | 0.791% | 368 | 73,162 | 0.503% | 1.57 | 1.42 | 1.75 |
| Citalopram* | 52 | 6659 | 0.781% | 98 | 18,245 | 0.537% | 1.45 | 1.19 | 1.78 |
| Escitalopram* | 37 | 6000 | 0.617% | 77 | 19,667 | 0.392% | 1.58 | 1.26 | 1.98 |
| Sertraline* | 38 | 4084 | 0.930% | 93 | 17,784 | 0.523% | 1.78 | 1.44 | 2.19 |
| Paroxetine* | 19 | 1414 | 1.344% | 64 | 8884 | 0.720% | 1.87 | 1.44 | 2.41 |
| **SNRI*** | 139 | 11,109 | 1.251% | 280 | 45,421 | 0.616% | 2.03 | 1.80 | 2.29 |
| Duloxetine* | 27 | 3012 | 0.896% | 54 | 11,332 | 0.477% | 1.88 | 1.43 | 2.47 |
| Venlafaxine* | 111 | 8042 | 1.380% | 218 | 33,518 | 0.650% | 2.12 | 1.85 | 2.43 |
| **NaSSA*** | 107 | 22,059 | 0.485% | 282 | 41,122 | 0.686% | 0.71 | 0.63 | 0.80 |
| Mirtazapine* | 93 | 20,812 | 0.447% | 265 | 39,493 | 0.671% | 0.67 | 0.59 | 0.75 |
| **Tricyclic antidepressants*** | 141 | 10,442 | 1.350% | 382 | 19794 | 1.930% | 0.70 | 0.63 | 0.78 |
| Amitriptyline* | 48 | 2416 | 1.987% | 99 | 11673 | 0.848% | 2.34 | 1.91 | 2.88 |
| Doxepin | 11 | 2416 | 0.455% | 60 | 11395 | 0.527% | 0.86 | 0.67 | 1.12 |
| Trimipramine* | 18 | 1979 | 0.910% | 74 | 11626 | 0.637% | 1.43 | 1.13 | 1.81 |
| Clomipramine | 17 | 1073 | 1.584% | 82 | 5102 | 1.607% | 0.99 | 0.78 | 1.25 |
| Nortriptyline | 20 | 1081 | 1.850% | 19 | 1454 | 1.307% | 1.42 | 0.89 | 2.24 |
| **Other antidepressant drugs*** | 38 | 4966 | 0.765% | 106 | 44,365 | 0.239% | 3.20 | 2.64 | 3.89 |
| Trazodone* | 17 | 2463 | 0.690% | 35 | 10108 | 0.346% | 1.99 | 1.42 | 2.79 |
| **Antipsychotic drugs*** | 606 | 70,325 | 0.862% | 2985 | 262,850 | 1.136% | 0.76 | 0.73 | 0.79 |
| **Low potency first-generation antipsychotic drugs*** | 142 | 27,320 | 0.520% | 327 | 75,149 | 0.435% | 1.19 | 1.07 | 1.33 |
| Pipamperone | 51 | 10,004 | 0.510% | 66 | 14,113 | 0.468% | 1.09 | 0.85 | 1.39 |
| Melperone* | 37 | 9623 | 0.384% | 19 | 9361 | 0.203% | 1.89 | 1.21 | 2.97 |
| Chlorprothixene* | 7 | 1089 | 0.643% | 63 | 12,929 | 0.487% | 1.32 | 1.02 | 1.71 |
| Prothipendyl* | 28 | 4426 | 0.632% | 29 | 11,316 | 0.256% | 2.47 | 1.71 | 3.56 |
| Levomepromazine* | 11 | 818 | 0.013% | 99 | 12,627 | 0.008% | 1.72 | 1.38 | 2.14 |
| Promethazine* | 14 | 1883 | 0.743% | 59 | 15,662 | 0.377% | 1.97 | 1.52 | 2.57 |
| **High potency first-generation antipsychotic drugs** | 144 | 15,241 | 0.945% | 707 | 77,739 | 0.909% | 1.04 | 0.96 | 1.12 |
| Haloperidol | 83 | 8630 | 0.962% | 312 | 29,020 | 1.075% | 0.89 | 0.80 | 1.00 |
| Flupentixol* | 11 | 1001 | 1.099% | 67 | 9822 | 0.682% | 1.61 | 1.25 | 2.08 |
| Perazine | 15 | 1375 | 1.091% | 133 | 14,121 | 0.942% | 1.16 | 0.96 | 1.40 |
| **Second-generation antipsychotic drugs*** | 426 | 44,245 | 0.963% | 2360 | 181,916 | 1.297% | 0.74 | 0.71 | 0.77 |
| Clozapine* | 76 | 3878 | 1.960% | 510 | 34,471 | 1.480% | 1.32 | 1.20 | 1.46 |
| Olanzapine* | 89 | 9353 | 0.952% | 633 | 45,469 | 1.392% | 0.68 | 0.63 | 0.74 |
| Quetiapine | 106 | 15,066 | 0.704% | 394 | 51,143 | 0.770% | 0.91 | 0.82 | 1.01 |
| Risperidone* | 117 | 14,923 | 0.784% | 465 | 36,760 | 1.265% | 0.62 | 0.56 | 0.68 |
| Amisulpride | 18 | 1152 | 1.562% | 205 | 13,016 | 1.575% | 0.99 | 0.85 | 1.16 |
| Aripiprazole | 11 | 1240 | 0.887% | 137 | 14,748 | 0.929% | 0.95 | 0.79 | 1.15 |
| **Traquilizing drugs*** | 56 | 29,854 | 0.188% | 110 | 113,252 | 0.097% | 1.93 | 1.60 | 2.33 |
| Lorazepam* | 31 | 19278 | 0.161% | 51 | 65,979 | 0.077% | 2.08 | 1.58 | 2.74 |
| Diazepam* | 16 | 4427 | 0.361% | 43 | 31,579 | 0.136% | 2.65 | 1.96 | 3.59 |
| **Hypnotic drugs*** | 22 | 14192 | 0.155% | 26 | 37,634 | 0.069% | 2.24 | 1.53 | 3.30 |
| **Antiepileptic drugs** | 143 | 19763 | 0.724% | 560 | 80,083 | 0.699% | 1.03 | 0.95 | 1.13 |
| Carbamazepine* | 39 | 3520 | 1.108% | 195 | 20,788 | 0.938% | 1.18 | 1.02 | 1.37 |
| Valproate* | 42 | 8272 | 0.508% | 213 | 33,987 | 0.627% | 0.81 | 0.71 | 0.93 |
| Lamotrigine | 13 | 2336 | 0.557% | 52 | 9687 | 0.537% | 1.04 | 0.78 | 1.37 |
| Pregabalin* | 32 | 3536 | 0.905% | 55 | 9448 | 0.582% | 1.55 | 1.19 | 2.03 |
| **Lithium*** | 73 | 5260 | 1.388% | 216 | 27,113 | 0.797% | 1.74 | 1.52 | 2.00 |
| **Antiparkinson drugs*** | 52 | 9941 | 0.523% | 87 | 35,186 | 0.247% | 2.12 | 1.71 | 2.62 |
| Biperiden* | 30 | 3790 | 0.792% | 70 | 29,433 | 0.238% | 3.33 | 2.62 | 4.23 |

*indicates a significant result

**N:** number (of); **LL:** lower limit; **UL:** upper limit; **SSRI:** selective serotonin reuptake inhibitor; **SNRI**: selective serotonin-norepinephrine reuptake inhibitor; **NaSSA:** noradrenergic and specific serotonergic antidepressant
