# Supplementary material for: Psychotropic drug-induced adverse drug reactions in 462,661 psychiatric inpatients in relation to age: results from a German drug surveillance program from 1993–2016
Source: Ann Gen Psychiatry. 2024 Nov 18;23:47. doi: 10.1186/s12991-024-00530-0 (PMC11575432; doi:10.1186/s12991-024-00530-0)
Supplement: Supplementary file 6 — Supplementary Table 6 [file 12991_2024_530_MOESM6_ESM.docx]

**Suppl. Table 6:** Relative risk (including 95% confidence interval) and incidence (in % of patients exposed to the respective drug/drug group of single imputation adverse drug reactions of psychotropic drugs and drug groups according to age (≥ 65 and < 65 years)

| **Drug/drug group** | **Patients ≥65 years of age** | | | **Patients <65 years of age** | | | **Relative Risk (older vs. younger patients)** | | |
| --- | --- | --- | --- | --- | --- | --- | --- | --- | --- |
|  | **N cases of ADRs and number of patients exposed to respective drug (group)** | **N patients exposed to respective drug (group)** | **% of patients exposed to drug (group) with ADR** | **N cases of ADRs and number of patients exposed to respective drug (group)** | **N patients exposed to respective drug (group)** | **% of patients exposed to drug (group) with ADR** | **RR** | **LL** | **UL** |
| **Any psychotropic drug*** | 592 | 99,099 | 0.597% | 2734 | 363,562 | 0.752% | 0.79 | 0.76 | 0.83 |
| **Antidepressant drugs** | 218 | 56,578 | 0.385% | 727 | 187,010 | 0.389% | 0.99 | 0.92 | 1.07 |
| **SSRI** | 54 | 19,333 | 0.279% | 208 | 73,162 | 0.284% | 0.98 | 0.86 | 1.13 |
| Citalopram* | 16 | 6659 | 0.240% | 60 | 18,245 | 0.329% | 0.73 | 0.57 | 0.94 |
| Escitalopram | 11 | 6000 | 0.183% | 48 | 19,667 | 0.244% | 0.75 | 0.56 | 1.00 |
| Sertraline* | 22 | 4084 | 0.539% | 62 | 17,784 | 0.349% | 1.55 | 1.20 | 1.99 |
| Paroxetine | 5 | 1414 | 0.354% | 38 | 8884 | 0.428% | 0.83 | 0.60 | 1.15 |
| **SNRI*** | 53 | 11,109 | 0.477% | 166 | 45,421 | 0.365% | 1.31 | 1.12 | 1.52 |
| Duloxetine | 9 | 3012 | 0.299% | 39 | 11,332 | 0.344% | 0.87 | 0.63 | 1.19 |
| Venlafaxine* | 44 | 8042 | 0.547% | 127 | 33,518 | 0.379% | 1.44 | 1.21 | 1.72 |
| **NaSSA*** | 50 | 22,059 | 0.227% | 161 | 41,122 | 0.392% | 0.58 | 0.50 | 0.68 |
| Mirtazapine* | 42 | 20,812 | 0.202% | 153 | 39,493 | 0.387% | 0.52 | 0.44 | 0.61 |
| **Tricyclic antidepressants*** | 58 | 10,442 | 0.555% | 180 | 19,794 | 0.909% | 0.61 | 0.53 | 0.71 |
| Amitriptyline* | 21 | 2416 | 0.869% | 51 | 11,673 | 0.437% | 1.99 | 1.50 | 2.63 |
| Doxepin | 6 | 2416 | 0.248% | 29 | 11,395 | 0.254% | 0.98 | 0.67 | 1.41 |
| Trimipramine | 6 | 1979 | 0.303% | 35 | 11,626 | 0.301% | 1.01 | 0.72 | 1.41 |
| Clomipramine | 6 | 1073 | 0.559% | 36 | 5102 | 0.706% | 0.79 | 0.56 | 1.11 |
| Nortriptyline | 8 | 1081 | 0.740% | 12 | 1454 | 0.825% | 0.90 | 0.50 | 1.59 |
| **Other antidepressant drugs*** | 3 | 4966 | 0.060% | 15 | 44,365 | 0.027% | 1.79 | 1.08 | 2.97 |
| Trazodone | 3 | 2463 | 0.122% | 12 | 10,108 | 0.119% | 1.03 | 0.58 | 1.81 |
| **Antipsychotic drugs*** | 260 | 70,325 | 0.370% | 1668 | 262,850 | 0.635% | 0.58 | 0.55 | 0.61 |
| **Low potency first-generation antipsychotic drugs*** | 29 | 27,320 | 0.106% | 50 | 75,149 | 0.067% | 1.60 | 1.21 | 2.11 |
| Pipamperone | 17 | 10,004 | 0.170% | 21 | 14,113 | 0.149% | 1.14 | 0.74 | 1.75 |
| Melperone | 6 | 9623 | 0.062% | 3 | 9361 | 0.032% | 1.95 | 0.63 | 6.04 |
| Chlorprothixene | 0 | 1089 | 0.000% | 12 | 12,929 | 0.093% | 0.00 | – | – |
| Prothipendyl* | 7 | 4426 | 0.158% | 2 | 11,316 | 0.062% | 8.95 | 2.24 | 35.81 |
| Levomepromazine* | 2 | 818 | 0.013% | 14 | 12,627 | 0.008% | 2.21 | 1.29 | 3.76 |
| Promethazine* | 4 | 1883 | 0.212% | 7 | 15,662 | 0.045% | 4.75 | 2.26 | 10.00 |
| **High potency first-generation antipsychotic drugs** | 41 | 15,241 | 0.269% | 218 | 77,739 | 0.280% | 0.96 | 0.84 | 1.10 |
| Haloperidol* | 32 | 8630 | 0.371% | 133 | 29,020 | 0.458% | 0.81 | 0.68 | 0.96 |
| Flupentixol* | 5 | 1001 | 0.500% | 27 | 9822 | 0.275% | 1.82 | 1.23 | 2.68 |
| Perazine* | 4 | 1375 | 0.291% | 58 | 14,121 | 0.411% | 0.71 | 0.54 | 0.93 |
| **Second-generation antipsychotic drugs*** | 190 | 44,245 | 0.429% | 1400 | 181,916 | 0.770% | 0.56 | 0.53 | 0.59 |
| Clozapine* | 47 | 3878 | 1.212% | 249 | 34,471 | 0.722% | 1.68 | 1.47 | 1.92 |
| Olanzapine* | 36 | 9353 | 0.385% | 385 | 45,469 | 0.847% | 0.45 | 0.41 | 0.50 |
| Quetiapine* | 38 | 15,066 | 0.252% | 185 | 51,143 | 0.362% | 0.70 | 0.60 | 0.81 |
| Risperidone* | 52 | 14,923 | 0.348% | 262 | 36,760 | 0.713% | 0.49 | 0.43 | 0.55 |
| Amisulpride | 12 | 1152 | 1.042% | 126 | 13,016 | 0.968% | 1.08 | 0.89 | 1.31 |
| Aripiprazole* | 5 | 1240 | 0.403% | 93 | 14,748 | 0.631% | 0.64 | 0.51 | 0.80 |
| **Traquilizing drugs*** | 9 | 29,854 | 0.030% | 11 | 113,252 | 0.010% | 3.10 | 1.72 | 5.61 |
| Lorazepam* | 7 | 19,278 | 0.036% | 10 | 65,979 | 0.015% | 2.40 | 1.29 | 4.45 |
| Diazepam | 1 | 4427 | 0.023% | 1 | 31,579 | 0.003% | 7.13 | 1.00 | 50.67 |
| **Hypnotic drugs** | 5 | 14,192 | 0.035% | 12 | 37,634 | 0.032% | 1.10 | 0.63 | 1.95 |
| **Antiepileptic drugs*** | 59 | 19,763 | 0.299% | 303 | 80,083 | 0.378% | 0.79 | 0.70 | 0.88 |
| Carbamazepine* | 18 | 3520 | 0.511% | 147 | 20,788 | 0.707% | 0.72 | 0.61 | 0.86 |
| Valproate* | 17 | 8272 | 0.206% | 87 | 33,987 | 0.256% | 0.80 | 0.65 | 0.99 |
| Lamotrigine | 8 | 2336 | 0.342% | 41 | 9687 | 0.423% | 0.81 | 0.59 | 1.11 |
| Pregabalin* | 16 | 3536 | 0.452% | 28 | 9448 | 0.296% | 1.53 | 1.05 | 2.22 |
| **Lithium*** | 17 | 5260 | 0.323% | 51 | 27,113 | 0.188% | 1.72 | 1.30 | 2.27 |
| **Antiparkinson drugs*** | 9 | 9941 | 0.091% | 11 | 35,186 | 0.031% | 2.90 | 1.60 | 5.23 |
| Biperiden* | 8 | 3790 | 0.211% | 10 | 29,433 | 0.034% | 6.21 | 3.34 | 11.57 |

*indicates a significant result

**N:** number (of); **LL:** lower limit; **UL:** upper limit; **SSRI:** selective serotonin reuptake inhibitor; **SNRI**: selective serotonin-norepinephrine reuptake inhibitor; **NaSSA:** noradrenergic and specific serotonergic antidepressant
