# Supplementary material for: Psychotropic drug-induced adverse drug reactions in 462,661 psychiatric inpatients in relation to age: results from a German drug surveillance program from 1993–2016
Source: Ann Gen Psychiatry. 2024 Nov 18;23:47. doi: 10.1186/s12991-024-00530-0 (PMC11575432; doi:10.1186/s12991-024-00530-0)
Supplement: Supplementary file 7 — Supplementary Table 7 [file 12991_2024_530_MOESM7_ESM.docx]

**Suppl. Table 7:** Relative risk (including 95% confidence interval) and incidence (in % of patients exposed to the respective drug/drug group of multiple imputation adverse drug reactions of psychotropic drugs and drug groups according to age (≥ 65 and < 65 years)

| **Drug/drug group** | **Patients ≥65 years of age** | | | **Patients <65 years of age** | | | **Relative Risk (older vs. younger patients)** | | |
| --- | --- | --- | --- | --- | --- | --- | --- | --- | --- |
|  | **N cases of ADRs and number of patients exposed to respective drug (group)** | **N patients exposed to respective drug (group)** | **% of patients exposed to drug (group) with ADR** | **N cases of ADRs and number of patients exposed to respective drug (group)** | **N patients exposed to respective drug (group)** | **% of patients exposed to drug (group) with ADR** | **RR** | **LL** | **UL** |
| **Any psychotropic drug*** | 620 | 99,099 | 0.626% | 1783 | 363,562 | 0.490% | 1.28 | 1.22 | 1.34 |
| **Antidepressant drugs*** | 320 | 56,578 | 0.566% | 614 | 187,010 | 0.328% | 1.72 | 1.59 | 1.87 |
| **SSRI*** | 99 | 19,333 | 0.512% | 160 | 73,162 | 0.219% | 2.34 | 2.00 | 2.74 |
| Citalopram* | 36 | 6659 | 0.541% | 38 | 18,245 | 0.208% | 2.60 | 1.88 | 3.57 |
| Escitalopram* | 26 | 6000 | 0.433% | 29 | 19,667 | 0.147% | 2.94 | 2.04 | 4.24 |
| Sertraline* | 16 | 4084 | 0.392% | 31 | 17,784 | 0.174% | 2.25 | 1.58 | 3.21 |
| Paroxetine* | 14 | 1414 | 0.990% | 26 | 8884 | 0.293% | 3.38 | 2.29 | 5.01 |
| **SNRI*** | 86 | 11,109 | 0.774% | 114 | 45,421 | 0.251% | 3.08 | 2.56 | 3.71 |
| Duloxetine* | 18 | 3012 | 0.598% | 15 | 11,332 | 0.132% | 4.51 | 2.71 | 7.51 |
| Venlafaxine* | 67 | 8042 | 0.833% | 91 | 33,518 | 0.271% | 3.07 | 2.49 | 3.78 |
| **NaSSA** | 57 | 22,059 | 0.258% | 121 | 41,122 | 0.294% | 0.88 | 0.73 | 1.05 |
| Mirtazapine | 51 | 20,812 | 0.245% | 112 | 39,493 | 0.284% | 0.86 | 0.72 | 1.04 |
| **Tricyclic antidepressants*** | 83 | 10,442 | 0.795% | 202 | 19,794 | 1.021% | 0.78 | 0.68 | 0.90 |
| Amitriptyline* | 27 | 2416 | 1.118% | 48 | 11,673 | 0.411% | 2.72 | 2.04 | 3.63 |
| Doxepin | 5 | 2416 | 0.207% | 31 | 11,395 | 0.272% | 0.76 | 0.53 | 1.09 |
| Trimipramine* | 12 | 1979 | 0.606% | 39 | 11,626 | 0.335% | 1.81 | 1.31 | 2.49 |
| Clomipramine | 11 | 1073 | 1.025% | 46 | 5102 | 0.902% | 1.14 | 0.84 | 1.54 |
| Nortriptyline* | 12 | 1081 | 1.110% | 7 | 1454 | 0.481% | 2.31 | 1.09 | 4.87 |
| **Other antidepressant drugs*** | 35 | 4966 | 0.705% | 91 | 44,365 | 0.000% | 3.44 | 2.79 | 4.24 |
| Trazodone* | 14 | 2463 | 0.568% | 23 | 10,108 | 0.228% | 2.50 | 1.65 | 3.78 |
| **Antipsychotic drugs** | 346 | 70,325 | 0.492% | 1317 | 262,850 | 0.501% | 0.98 | 0.93 | 1.04 |
| **Low potency first-generation antipsychotic drugs** | 113 | 27,320 | 0.414% | 277 | 75,149 | 0.369% | 1.12 | 1.00 | 1.26 |
| Pipamperone | 34 | 10,004 | 0.340% | 45 | 14,113 | 0.319% | 1.07 | 0.79 | 1.43 |
| Melperone* | 31 | 9623 | 0.322% | 16 | 9361 | 0.171% | 1.88 | 1.15 | 3.08 |
| Chlorprothixene* | 7 | 1089 | 0.643% | 51 | 12,929 | 0.394% | 1.63 | 1.22 | 2.17 |
| Prothipendyl* | 22 | 4426 | 0.497% | 26 | 11,316 | 0.230% | 2.16 | 1.47 | 3.19 |
| Levomepromazine* | 9 | 818 | 1.100% | 85 | 12,627 | 0.673% | 1.63 | 1.29 | 2.07 |
| Promethazine* | 10 | 1883 | 0.531% | 52 | 15,662 | 0.332% | 1.60 | 1.21 | 2.12 |
| **High potency first-generation antipsychotic drugs** | 103 | 15,241 | 0.676% | 489 | 77,739 | 0.629% | 1.07 | 0.98 | 1.18 |
| Haloperidol | 51 | 8630 | 0.591% | 179 | 29,020 | 0.617% | 0.96 | 0.82 | 1.11 |
| Flupentixol* | 6 | 1001 | 0.599% | 40 | 9822 | 0.407% | 1.47 | 1.07 | 2.03 |
| Perazine* | 11 | 1375 | 0.800% | 75 | 14,121 | 0.531% | 1.51 | 1.19 | 1.91 |
| **Second-generation antipsychotic drugs** | 236 | 44,245 | 0.533% | 960 | 181,916 | 0.528% | 1.01 | 0.95 | 1.08 |
| Clozapine | 29 | 3878 | 0.748% | 261 | 34,471 | 0.757% | 0.99 | 0.87 | 1.12 |
| Olanzapine | 53 | 9353 | 0.567% | 248 | 45,469 | 0.545% | 1.04 | 0.91 | 1.18 |
| Quetiapine | 68 | 15,066 | 0.451% | 209 | 51,143 | 0.409% | 1.10 | 0.96 | 1.27 |
| Risperidone* | 65 | 14,923 | 0.436% | 203 | 36,760 | 0.552% | 0.79 | 0.69 | 0.91 |
| Amisulpride | 6 | 1152 | 0.521% | 79 | 13,016 | 0.607% | 0.86 | 0.68 | 1.09 |
| Aripiprazole* | 6 | 1240 | 0.484% | 44 | 14,748 | 0.298% | 1.62 | 1.19 | 2.20 |
| **Traquilizing drugs*** | 47 | 29,854 | 0.157% | 99 | 113,252 | 0.087% | 1.80 | 1.48 | 2.19 |
| Lorazepam* | 24 | 19,278 | 0.124% | 41 | 65,979 | 0.062% | 2.00 | 1.47 | 2.72 |
| Diazepam* | 15 | 4427 | 0.339% | 42 | 31,579 | 0.133% | 2.55 | 1.88 | 3.46 |
| **Hypnotic drugs*** | 17 | 14192 | 0.120% | 14 | 37,634 | 0.037% | 3.22 | 1.91 | 5.44 |
| **Antiepileptic drugs*** | 84 | 19763 | 0.425% | 257 | 80,083 | 0.321% | 1.32 | 1.17 | 1.50 |
| Carbamazepine* | 21 | 3520 | 0.597% | 48 | 20,788 | 0.231% | 2.58 | 1.94 | 3.44 |
| Valproate* | 25 | 8272 | 0.302% | 126 | 33,987 | 0.371% | 0.82 | 0.68 | 0.97 |
| Lamotrigine* | 5 | 2336 | 0.214% | 11 | 9687 | 0.114% | 1.88 | 1.04 | 3.41 |
| Pregabalin* | 16 | 3536 | 0.452% | 27 | 9448 | 0.286% | 1.58 | 1.08 | 2.32 |
| **Lithium*** | 56 | 5260 | 1.065% | 165 | 27,113 | 0.609% | 1.75 | 1.49 | 2.05 |
| **Antiparkinson drugs*** | 43 | 9941 | 0.433% | 76 | 35,186 | 0.216% | 2.00 | 1.60 | 2.51 |
| Biperiden* | 22 | 3790 | 0.580% | 60 | 29,433 | 0.204% | 2.85 | 2.20 | 3.68 |

*indicates a significant result

**N:** number (of); **LL:** lower limit; **UL:** upper limit; **SSRI:** selective serotonin reuptake inhibitor; **SNRI**: selective serotonin-norepinephrine reuptake inhibitor; **NaSSA:** noradrenergic and specific serotonergic antidepressant
